# VIS–NIR–SWIR Hyperspectral Imaging and Advanced Machine and Deep Learning Algorithms for a Controlled Benchmark of Bean Seed Identification and Classification

**DOI:** 10.3390/plants15060933

**Published:** 2026-03-18

**Authors:** Renan Falcioni, Nicole Ghinzelli Vedana, Caio Almeida de Oliveira, João Vitor Ferreira Gonçalves, Marcelo Luiz Chicati, José Alexandre M. Demattê, Marcos Rafael Nanni

**Affiliations:** 1 Graduate Program in Agronomy, State University of Maringá, Av. Colombo 5790, Maringá 87020-900, Paraná, Brazil; pg405864@uem.br (N.G.V.); pg55482@uem.br (C.A.d.O.); pg55494@uem.br (J.V.F.G.); mlchicati@uem.br (M.L.C.); mrnanni@uem.br (M.R.N.); 2 Department of Soil Science, Luiz de Queiroz College of Agriculture, University of São Paulo, Av. Pádua Dias 11, Piracicaba 13418-260, São Paulo, Brazil; jamdemat@usp.br

**Keywords:** Aisa-FENIX sensor, deep learning, discriminant analysis, germplasm conservation, hyperspectral imaging, *Phaseolus vulgaris*, seed authentication

## Abstract

Reliable seed accession identification underpins germplasm conservation, traceability and breeding; however, conventional assays remain destructive, labour-intensive and difficult to scale. Here, visible–near-infrared–shortwave infrared (VIS–NIR–SWIR) hyperspectral imaging (HSI; 449.54–2399.17 nm; 563 bands) was used to classify 32 grain–legume accessions (*n* = 3200 seeds; 100 seeds per accession), comprising 30 common bean (*Phaseolus vulgaris* L.) landraces plus two outgroup legumes (*Vigna angularis* (Willd.) Ohwi & Ohashi and *Cajanus cajan* (L.) Huth). Each seed was represented by one ROI-averaged spectrum obtained from mean representative pixels within a standardised 10 × 10 pixel window at the centre of each seed. A fixed stratified 70:30 seed-level training:test partition was used, with 70 seeds per accession (*n* = 2240) reserved for fully independent training and 30 seeds per accession (*n* = 960) reserved as a fully independent test set. Principal component analysis (PCA) captured 97.42% of the spectral variance in the first three components (PC1 = 63.34%, PC2 = 23.78%, and PC3 = 10.31%). One-versus-rest wavelength association mapping revealed a maximum R^2^ of 0.775 at 461.37 nm, and ReliefF concentrated the strongest reduced-band signal within 449.54–456.30 nm and 577.02–597.54 nm. In the original ReliefF-selected 16-band benchmark, the subspace discriminant reached 68.25% macro-F1 and 68.54% balanced accuracy; after edge-band trimming, the alternative 16-band configuration decreased to 60.67% and 60.94%, respectively. With respect to the full-spectrum sensitivity benchmark, linear discriminant analysis achieved 96.35% balanced accuracy, followed by linear SVM (94.17%). Deep learning trained directly on the full 563-band spectra reached 84.90% test accuracy, 84.47% macro-F1, 86.27% precision and 84.90% recall, with MLP_Wide outperforming the convolutional, recurrent and attention-based alternatives. Overall, under controlled laboratory conditions, this benchmark shows that accession discrimination is driven mainly by visible-domain contrasts in the most compact representations, whereas the full spectral context remains important for the most confusable accessions and for cautious future sensor design. The reduced-band findings should therefore be interpreted as exploratory guidance for sensor design rather than as a validated deployment-ready specification.

## 1. Introduction

Delivering sustainable gains in food and nutrition under climate constraints is increasingly limited by measurement capacity [1,2]. In seed-based systems, the ability to quantify and track biologically meaningful variation quickly, non-destructively and at scale is essential for conserving agrobiodiversity, supporting breeding decisions and safeguarding seed supply chains [1,2,3].

Common bean (*Phaseolus vulgaris* L.) is central to this agricultural agenda because it is simultaneously a global staple and a reservoir of adaptive diversity that can be mobilised for resilience and quality [4,5]. Recent synthesis work frames common bean improvement as a coupled challenge spanning climate stress [4], nutritional value and the need to leverage diversity across gene pools and associated microbiomes [4,5]. A comparable leverage of conserved diversity is also emphasised across other grain legumes, including soybean improvement pipelines, reinforcing the broader value of accession-level authentication in legume breeding [6]. Pangenome, seed colour and production belt analyses further reveal why identity, traceability and adaptive diversity must be resolved together in Brazilian bean systems [7,8,9].

Despite this importance, the characterisation of bean accessions and landraces remains operationally difficult because many materials share overlapping visual traits and because phenotype expression can be confounded by handling, ageing and heterogeneous acquisition conditions [10,11,12,13]. Traditional identification based on morphology or visual inspection is rapid but often ambiguous under subtle phenotypic differences, whereas genotyping is decisive yet rarely suited to routine, high-throughput screening in breeding stations, community seed banks or processing lines [7,14,15]. Recently, morphological and molecular studies of common bean germplasms have continued to report substantial diversity while also revealing how resource-intensive it can be to resolve that diversity with conventional descriptors alone [4,16], motivating artificial intelligence, machine learning and deep learning strategies that are both rigorous and scalable [4,16,17,18]. Similarly, seed-focused NIR/HSI (Near-Infrared/Hyperspectral Imaging) studies on aged or viable seed lots have shown that subtle phenotypic drift and handling history can reduce separability unless acquisition and validation are tightly standardised [19,20].

Hyperspectral imaging (HSI) offers a non-destructive route to close this gap because reflectance encodes pigment and surface structures in the visible range and extends into the NIR–SWIR (Near-Infrared–Short-Wave Infrared) region, where absorption is related to macromolecular composition in several species [13,21,22,23,24]. However, recent reviews emphasise persistent barriers: hyperspectral datasets are high-dimensional and strongly redundant, labelled samples are often limited, and their predictive performance can degrade under shifts in sensors and acquisition conditions [13,16,23,24,25]. The wider imaging spectroscopy landscape reinforces this need for wavelength-stable, transferable reasoning across platforms, as illustrated by early EnMAP (Environmental Mapping and Analysis Program) results [26]. In parallel, agricultural machine learning has moved beyond accuracy-first reporting; work in artificial intelligence in agriculture and recent explainable-AI reviews argue that interpretability, wavelength attribution and deployability constraints are prerequisites for trustworthy, decision-relevant modelling [17,27,28,29]. The seed imaging literature is also expanding across legumes and cereals, including soybean spectral unmixing and variety identification pipelines in common bean, maize/corn, grapevine, rice, tobacco, tomato, soybean and many other crops, where reported gains remain strongly protocol dependent [30,31,32,33,34,35,36]. Recent seed variety identification studies have therefore explored 1D CNN/ResNet-style architectures and related deep learning pipelines for hyperspectral signatures, often reporting strong performance under controlled acquisition settings. However, validation protocols vary widely, motivating the present study’s fixed-seed-level split and cross-family benchmarking under identical evaluation constraints.

Despite rapid progress in HSI-based seed authentication, practical gaps remain for germplasm-scale use: model comparisons are often difficult to interpret when validation protocols differ, and accuracy-first reporting rarely connects discrimination to wavelength-level attribution and computational feasibility [17,27,28,29]. A traceable benchmark that couples consistent seed-level validation with interpretable spectral attribution is therefore valuable for both scientific understanding and deployable sensing. This finding is consistent with recent surveys of hyperspectral deep learning, transformers in remote sensing, dimensionality reduction, band selection strategies and resolution/SNR (Signal-to-Noise Ratio) effects, all of which argue that architecture choice, feature compression and sensor design must be treated as coupled decisions rather than as isolated modelling steps [37,38,39,40].

Accordingly, this work contributes the following: (i) a traceable VIS–NIR–SWIR seed-level benchmark for 32 grain–legume accessions (*n* = 3200; 563 analysed bands spanning 449.54–2399.17 nm); (ii) a fixed stratified 70:30 seed-level training:test partition, with the same independent test set reused across spectral representations and model families; (iii) an interpretable analysis that links low-dimensional variance structure (PCA) and wavelength attribution (one-versus-rest association mapping, ReliefF, permutation importance and SHAP; SHapley Additive exPlanations) to classification outcomes; and (iv) computational diagnostics (training time, throughput and model size) to support deployability decisions. The present study is intentionally positioned as a controlled benchmark, and robustness to non-ideal acquisition conditions remains to be quantified.

Here, we present a traceable VIS–NIR–SWIR hyperspectral benchmark of thirty-two grain–legume accessions spanning phenotypes relevant to Brazilian agrobiodiversity, including seed lots that are visually distinct and seed lots that are visually confusable. We quantify how reliably ROI-level (Region of Interest) reflectance signatures support accession identification and benchmark classical machine learning and deep learning algorithms under a consistent, stratified seed-level 70:30 split. PCA is used explicitly as a mechanistic bridge between sensor space and interpretation, linking dominant modes of spectral variance to coherent wavelength regions and testing whether those regions align with the spectral windows that drive predictive discrimination.

We hypothesise that accession separability is predominantly low-dimensional and concentrated within contiguous spectral windows. This would enable band-reduced representations compatible with lower-cost sensors. A further hypothesis is that, in the moderate-data regime typical of curated germplasm screening, relatively simple one-dimensional deep models are more reliable than more elaborate sequence architectures because their inductive biases better match the signal-to-sample ratio. We test these hypotheses by combining wavelength-resolved association analysis and supervised band prioritisation with systematic benchmarking of classical and deep learners, alongside explicit computational profiling, to translate statistical performance into practical feasibility for conservation, traceability and scalable agricultural sensing. Any translational implication for lower-cost sensing is therefore treated here as provisional and subject to explicit external validation.

## 2. Results

### 2.1. Phenotypic Diversity and Accession Benchmarks

**Figure 1:**
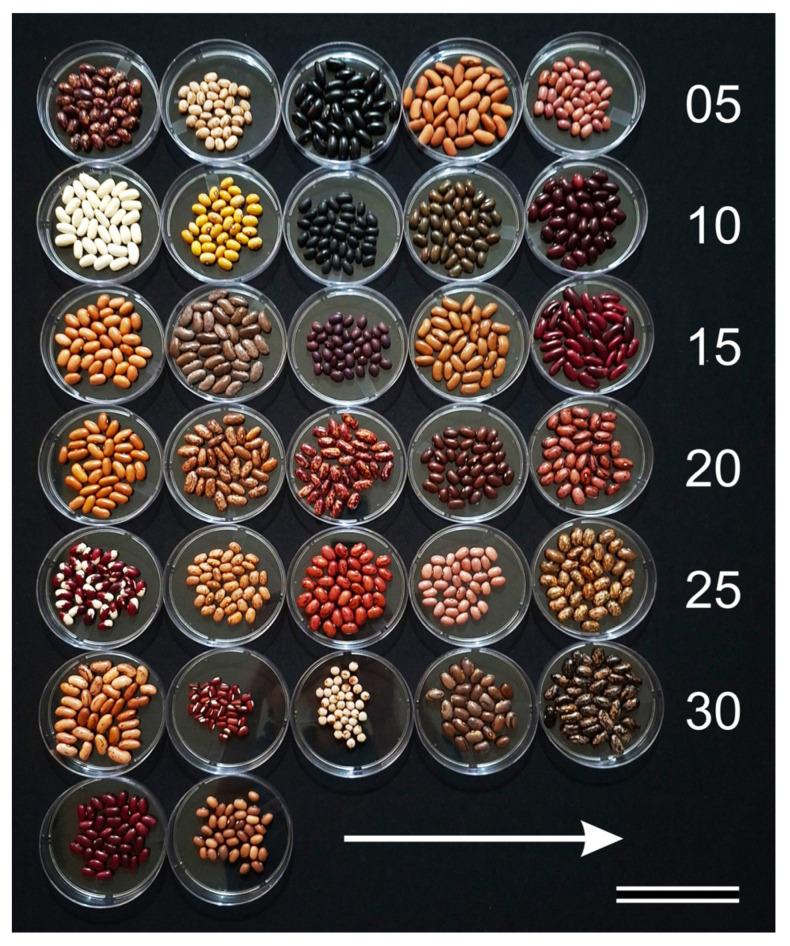
Seeds from the 32 grain–legume accessions were arranged in 10 cm Petri dishes prior to hyperspectral image acquisition. Dishes were organised sequentially from A01 to A32, left to right within each row (arrow), with the sequence restarting at the left of the next row (A01–A05, A06–A10, A11–A15, A16–A20, A21–A25 and A26–A30; A31–A32 in the final row). The numerals on the right indicate the last accession in each row. Accessions A01–A26 and A29–A32 are *Phaseolus vulgaris* L.; A27 is *Vigna angularis* (Willd.) Ohwi & H. Ohashi and A28 is *Cajanus cajan* (L.) Huth. Scale bar = 8.5 cm.

**Figure 2:**
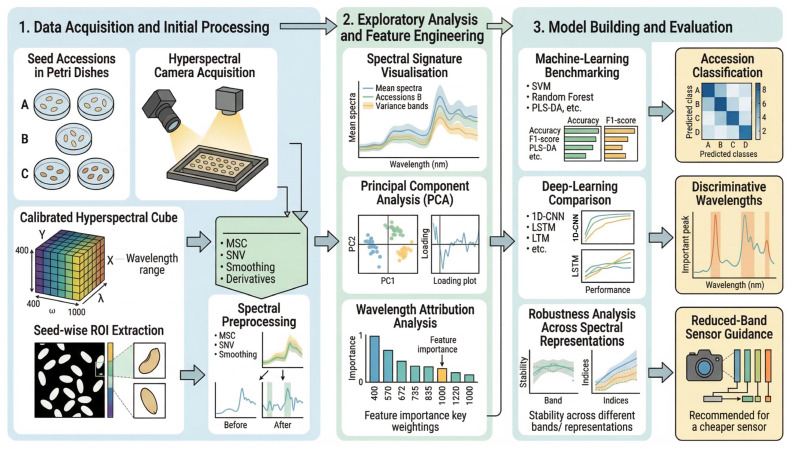
Workflow for hyperspectral seed accession identification and interpretable modelling. Seeds from 32 grain–legume accessions were scanned with an Aisa-FENIX hyperspectral sensor (Specim, Oulu, Northern Ostrobothnia, Finland); the analysed wavelength axis used in the present workflow comprised 563 bands spanning 449.54–2399.17 nm. After radiometric calibration (dark and Spectralon^®^ white reference), ROI-level mean spectra were extracted using a standardised 10 × 10 pixel ROI window per seed, from which 10 representative interior pixels were selected and averaged to yield one spectrum per seed (*n* = 3200). The spectra were preprocessed (Savitzky–Golay smoothing; clipping to [0, 1]; optional baseline correction and SNV), and exploratory plus interpretability analyses (PCA, one-versus-rest Pearson r/R^2^ mapping, hierarchical clustering, ReliefF, permutation importance and SHAP) were performed. A fixed stratified 70:30 seed-level training:test partition was used throughout. For the classical benchmark, the development subset was divided evenly into training and validation subsets (35:35:30 overall), whereas deep learning used an internal validation subset within the development portion and the 30% test set only for final evaluation. ReliefF identified the original 16-band windows as 449.54–456.30 nm and 577.02–597.54 nm; after edge-band trimming, the alternative 16-band configuration shifted to 575.31–600.96 nm.

The experimental set comprised thirty-two grain–legume accessions (A01–A32) whose coat colour, patterning and grain morphology spans a broad range of seed phenotypes (Figure 1), with accession codes retained throughout the workflow to preserve traceability across imaging (Figure 2), spectral extraction and modelling outputs (Table S1). The collection included uniformly pigmented coats spanning dark (black and deep brown), high-albedo (white and cream) and intermediate hues (pink, red, yellow and green tones), alongside patterned phenotypes including striped, mottled and bicolour seeds with localised hilum contrast. Morphological diversity further broadened the benchmark, ranging from small, rounded grains to large kidney-shaped and elongated “peanut-type” forms. In addition to common bean materials, the set intentionally included non-*Phaseolus* accessions (A27 and A28) to extend the explored phenotypic space within a single acquisition campaign. The analysed dataset comprised 3200 independent ROI-level spectra (100 per accession), providing a controlled yet heterogeneous benchmark in which visually separable and visually ambiguous accessions coexisted (Figure 1 and Figure 2). Each ROI-level spectrum corresponds to one seed and was obtained by averaging 10 representative pixels selected within a standardised 10 × 10 pixel ROI window, yielding exactly one spectrum per seed. Because the benchmark is intentionally spectral-only, we modelled accession identity using only 1D ROI-averaged reflectance signatures (no spatial texture/shape descriptors from the hyperspectral images were used).

### 2.2. Accession-Level Reflectance Structure Across the VIS–NIR–SWIR Range

**Figure 3:**
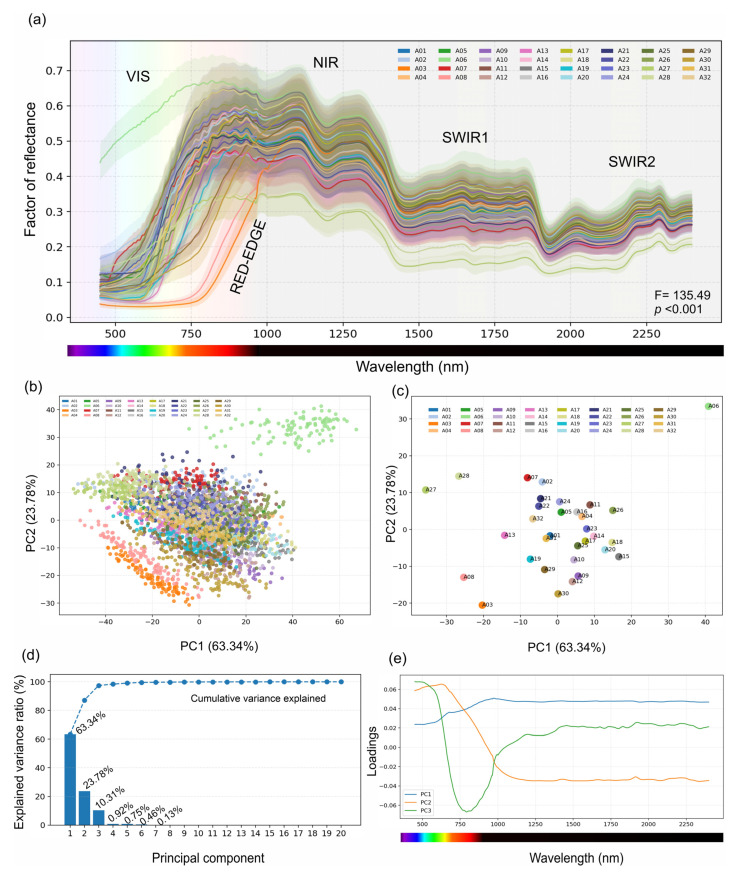
Spectral variability and PCA structure across 32 seed accessions based on preprocessed hyperspectral imaging data (449.54–2399.17 nm; 563 bands). (**a**) Mean reflectance spectra across accessions, with shaded regions indicating the VIS, red-edge transition, NIR, and SWIR1/SWIR2 ranges; PERMANOVA F = 135.49 (*p* < 0.001) summarises the overall effects of accession on reflectance. (**b**) PCA score plot for all spectra coloured by accession. (**c**) PCA score plot showing the accession centroids. (**d**) Explained variance (percentage) for the first components (PC1 = 63.34%, PC2 = 23.78%, PC3 = 10.31%; cumulative PC1–PC3 = 97.42%). (**e**) Loading profiles for PC1–PC3, highlighting wavelength contributions.

The mean reflectance spectra extracted from regions of interest exhibited pronounced differences across the measured VIS-NIR-SWIR domain (Figure 3a). In the visible range, the spectra were characterised by low reflectance with clear separation between accessions, followed by a steep transition through the red-edge region into the near-infrared region, where the reflectance rose sharply and stabilised into a broad plateau. Across the shortwave infrared, the reflectance decreased relative to that of the NIR plateau and displayed structured undulations with distinct absorption features that were consistently expressed but varied in magnitude among accessions. A06, corresponding to a high-albedo white seed coat phenotype, presented systematically elevated reflectance relative to the rest of the collection across much of the range, whereas several darker accessions retained lower reflectance in the visible domain. PERMANOVA confirmed statistically significant differences among the accessions (F = 135.49, *p* < 0.001; Figure 3a).

A preprocessing sensitivity analysis comparing the default Savitzky–Golay workflow with optional baseline correction and/or SNV did not alter the identity of the strongest full-spectrum classical models, supporting the main preprocessing scheme used throughout the manuscript (Figure 2 and Figure 3).

### 2.3. Multivariate Organisation of Spectral Variability via Principal Component Analysis

Principal component analysis (PCA) compressed the high-dimensional spectral matrix into a small number of dominant axes capturing most of the variance (Figure 3b–e). The first principal component (PC1) explained 63.34% of the total variance, PC2 explained 23.78%, and PC3 explained 10.31%, such that the first three components accounted for 97.42% of the spectral variability (Figure 3d). In the ROI-level score space, the spectra formed a continuous manifold with accession-specific shifts in position rather than strictly discrete clusters, indicating a partial overlap among subsets of accessions despite a substantial between-accession structure (Figure 3b). The class-mean centroids highlighted uneven separation in the PC1–PC2 plane (Figure 3c). A06 occupied an extreme region with large positive scores on both axes, whereas the non-*Phaseolus* accessions A27 and A28 were displaced strongly toward negative PC1 values, and A03 and A30 occupied the most negative PC2 region. The loading profiles indicated that these axes were driven by broad, contiguous wavelength contributions rather than isolated single-band effects, which is consistent with the large-scale albedo, visible absorption and longer-wavelength contrast structure.

### 2.4. Wavelength-Resolved Association Patterns, Spectral Similarity and Band Prioritisation

**Figure 4:**
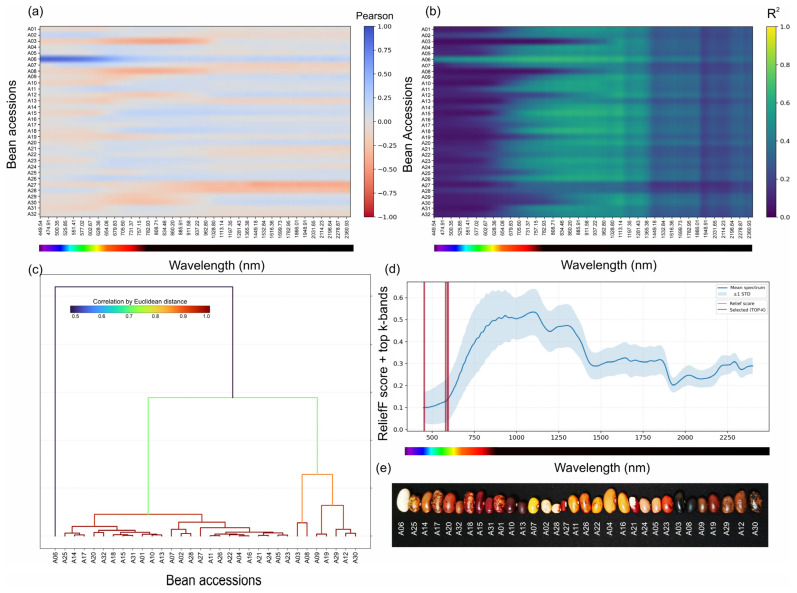
Correlation, spectral similarity and feature importance mapping across the 32 accessions. (**a**) Pearson correlation coefficients (r) between wavelength-specific reflectance values and one-versus-rest encoded accession labels (one row per accession; one column per wavelength). (**b**) Corresponding coefficient-of-determination (R^2^) map highlighting wavelength regions with strong one-versus-rest associations. (**c**) Hierarchical clustering dendrogram constructed from Euclidean distances between accession-mean spectra. (**d**) ReliefF feature importance profile across wavelengths; vertical lines indicate the 16 selected bands (449.54–456.30 nm and 577.02–597.54 nm). (**e**) Representative seed images arranged in the dendrogram leaf order to provide a visual reference for the spectral neighbourhoods (Aisa-FENIX sensor).

Wavelength-wise one-versus-rest association patterns demonstrated that class-related information was unevenly distributed across the spectrum (Figure 4a,b). The Pearson association maps revealed an accession-specific sign structure and wavelength-dependent magnitude changes, whereas the corresponding coefficient-of-determination representation highlighted localised regions of increased explanatory power rather than uniform separability. The strongest one-versus-rest association in the full dataset occurred at 461.37 nm (A06; R^2^ = 0.775), indicating that restricted visible regions can be highly informative even when collection-level separability remains heterogeneous.

Spectral similarity analysis based on Euclidean distances between accession-mean spectra yielded a hierarchical organisation in which most accessions formed a large coherent cluster, with a smaller set occupying distant branches (Figure 4c). In parallel, supervised band ranking via ReliefF converged on a compact subset of informative wavelengths concentrated in the visible domain. The 16 full-dataset selected bands occupied two windows (449.54–456.30 nm and 577.02–597.54 nm), five of which were edge bands. After edge-band trimming, the alternative 16-band configuration shifted to 575.31–600.96 nm and retained 11 wavelengths in common with the original set. Consistently, the accession-mean Pearson correlation matrix (Figure S1) revealed that many accession pairs remained highly correlated, including some with visually different seeds, indicating that pairwise similarity alone is insufficient for accession discrimination. Together, these patterns highlight a robust visible identity signal while also showing that supervised classifiers exploit subtle multiband structures more effectively than a simple correlation matrix does and that the exact reduced-band specification is sensitive to spectral-edge handling. A compact cross-representation summary is provided in Table 1, while the complementary accession–correlation structure, supervised ranking concordance and edge-band quality control are detailed in Figures S1–S3 and Table S2.

### 2.5. Accession Classification from Hyperspectral Signatures: Model Ranking and Confusion Structure

**Figure 5:**
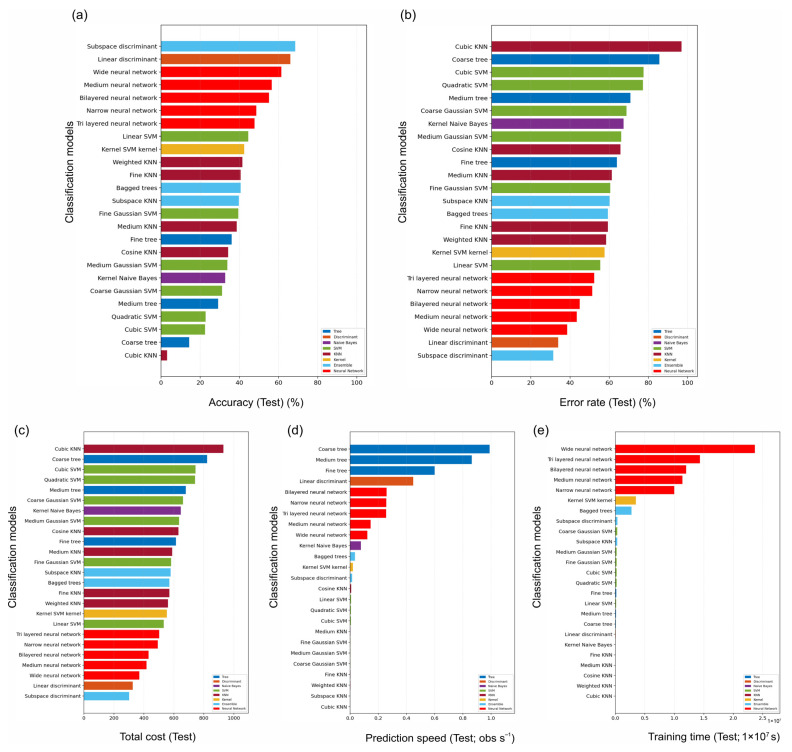
Test set performance and computational profiling across the full suite of multiclass classifiers trained on the original ReliefF-selected 16-band representation (including five edge bands). Panels summarise (**a**) test accuracy (%), (**b**) test error (%), (**c**) total misclassification cost, (**d**) prediction throughput (observations s^−1^) and (**e**) training time (s).

Multiclass accession classification on the original ReliefF-selected 16-band representation (including five edge bands) showed substantial variation across the 25 classical classifiers (Figure 5). Under the fixed 35:35:30 training:validation:test implementation of the overall 70:30 training:test protocol, the test accuracy ranged from 3.12% (Cubic KNN) to 68.54% (subspace discriminant). The strongest reduced-band model was subspace discriminant, which achieved 68.54% accuracy, 68.25% macro-F1 and 68.54% balanced accuracy. Linear discriminant analysis ranked second (66.04% accuracy), and the best shallow neural network (Wide neural network) reached 61.46% accuracy. Thus, the original compact visible-band representation retained clear accession information, but it did not reproduce the discrimination obtained from the full spectra. The strongest full-spectrum models are ranked separately in Table 2, and the accuracy and macro-F1 profiles of the additional baseline classifiers are summarised in Figure S7.

**Figure 6:**
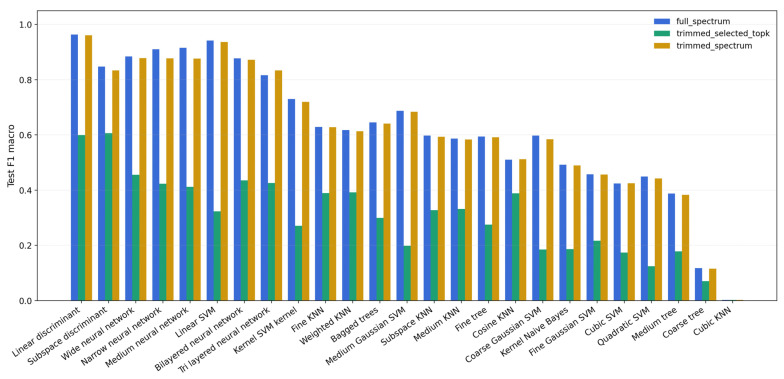
Sensitivity of classifier performance to spectral representation. All the evaluated models are compared using held-out macro-F1 on the full-spectrum (563-band), trimmed-spectrum (555-band) and trimmed-spectrum-plus-selected-top-k (edge-trimmed 16-band) representations, enabling direct assessment of how spectral compression redistributes the model ranking.

Spectral representation sensitivity clarified the trade-off between compactness and performance (Table 1; Figure 6 and Figure S11). In the full-spectrum benchmark, linear discriminant analysis reached 96.35% macro-F1 and 96.35% balanced accuracy, followed by linear SVM (94.17%/94.17%). Trimming the eight spectral-edge bands had a negligible effect on the best model (linear discriminant: 96.13% macro-F1; 96.15% balanced accuracy), whereas restricting the feature space to the edge-trimmed alternative 16-band representation lowered the best score to 60.67% macro-F1 and 60.94% balanced accuracy.

Row-normalised confusion matrices for the benchmark classifiers are provided in Figures S8 and S9, and the microaveraged one-versus-rest ROC and precision–recall curves for the principal benchmark models are summarised in Figure S10. These supplementary panels show that a strong diagonal structure is maintained only for the best full-spectrum models, whereas the reduced-band representation exhibits much broader off-diagonal dispersion and therefore a substantially more diffuse error structure. Numerical micro- and macroaveraged ROC-AUC and average precision summaries for these benchmark models are additionally reported in Table S3, whereas development-versus-test agreement and internal optimisation diagnostics are shown in Figures S4 and S5.

The spectral representation heatmap (Figure S11) reinforced this pattern across the full model suite. Nearly all of the best-performing classifiers benefitted from the complete 563-band spectra, whereas the 555-band edge-trimmed representation remained essentially equivalent for the strongest models. In contrast, the 16-band representation compressed the model ranking and lowered all the top scores, indicating that the highlighted visible windows are informative but not sufficient on their own to recover accession identity at full-spectrum fidelity.

The computational and storage characteristics also varied markedly in the reduced-band benchmark (Figure 5c–e). The total misclassification cost ranged from 302 (subspace discriminant) to 930 (Cubic KNN). The prediction throughput ranged from 1.55 × 10^4^ observations s^−1^ (subspace KNN) to 9.90 × 10^6^ observations s^−1^ (Coarse tree), whereas the training time ranged from 9.44 × 10^−4^ s (Cubic KNN) to 2.37 s (Wide neural network). Serialised model size ranged from 4.39 kB (Coarse tree) to 8.31 MB (subspace KNN), highlighting that deployability depends on far more than the predictive score alone.

### 2.6. Deep Learning Architectures: Learning Dynamics and Independent Test Set Performance

**Figure 7:**
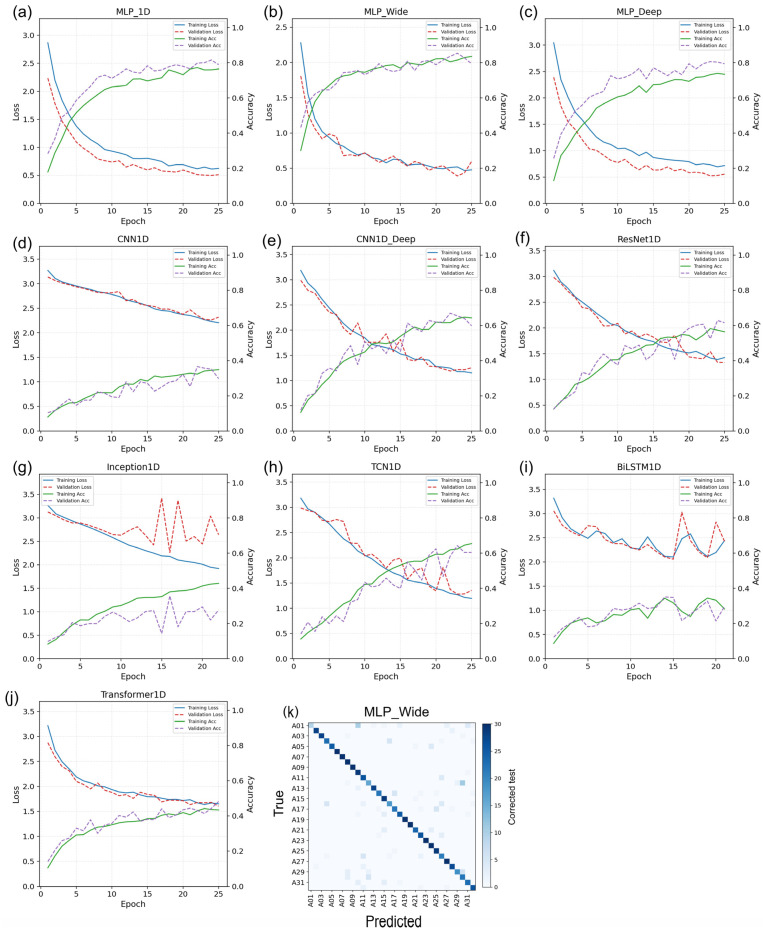
Training dynamics of one-dimensional deep learning architectures fitted to full-spectrum reflectance sequences. Panels (**a**–**j**) show the epoch-wise training and validation loss and accuracy. The blue solid line represents training loss, the red dashed line represents validation loss, the green solid line represents training accuracy, and the purple dashed line represents validation accuracy. Panel (**k**) shows the confusion matrix for the best-performing deep model (MLP_Wide) on the independent test set (counts; 30 test seeds per accession).

**Figure 8:**
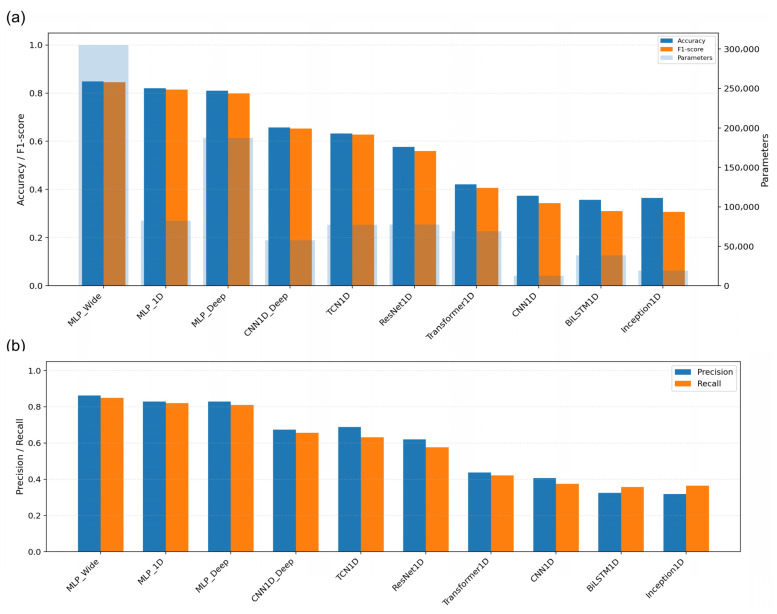
Summary performance and complexity of deep learning models on the accession classification task. (**a**) Comparison of accuracy and F1 score across architectures, jointly displayed with model size expressed as the number of trainable parameters (second axis). (**b**) Precision and recall for the same set of models, enabling assessment of error asymmetries (false positives versus false negatives) and highlighting architectures that maintain balanced sensitivity and specificity.

The deep learning models trained on the full 563-band reflectance sequences clearly differed in terms of both learning dynamics and generalisation (Table 3; Figure 7 and Figure 8). Among the ten one-dimensional architectures, MLP_Wide achieved the strongest independent test set performance (accuracy = 84.90%; F1 score = 84.47%; precision = 86.27%; recall = 84.90%), followed by MLP_1D (accuracy = 81.98%; F1 score = 81.38%) and MLP_Deep (accuracy = 80.94%; F1 score = 79.84%). The convolutional and temporal models formed a middle tier (CNN1D_Deep: 65.73% accuracy; TCN1D: 63.23%; ResNet1D: 57.60%), whereas Transformer1D, CNN1D, Inception1D and BiLSTM1D remained below 43% accuracy. Under a fixed training budget, the parameter count did not map monotonically onto held-out performance.

The training trajectories further differentiated the architectures (Figure 7). Compared with the recurrent and attention-based models, the three MLP variants converged rapidly, with smoother validation behaviour and lower sustained generalisation gaps. In contrast, several convolutional, residual, temporal and transformer-style architectures continued to optimise training loss after the validation performance plateaued, indicating that added sequential complexity was not advantageous for this moderate-sized one-dimensional spectral dataset (Figure 8).

## 3. Discussion

### 3.1. What Do the Spectra Say About Accession Identity?

Across the analysed 449.54–2399.17 nm range, the accession-level reflectance signatures were internally coherent within classes, but the separability was strongly wavelength dependent. The strongest one-versus-rest signal occurred at 461.37 nm (R^2^ = 0.775), and ReliefF concentrated the exploratory reduced-band signal within two visible windows: 449.54–456.30 nm and 577.02–597.54 nm. This pattern is mechanistically consistent with the fact that seed coat pigmentation, local scattering and testa surface properties dominate shortwave reflectance contrasts among visually distinct landraces.

A practical corollary is that, in this benchmark, accession identity is expressed primarily through testa-level optical phenotypes rather than through the longer-wave absorptions typically emphasised for bulk chemical prediction. The NIR–SWIR region still contributes materially to full-spectrum classification, but its role appears complementary rather than dominant for accession identity under the controlled acquisition conditions used here.

Importantly, five of the 16 full-dataset ReliefF bands were edge bands. When these were removed from the trimmed analysis, the alternative 16-band configuration shifted to 575.31–600.96 nm while retaining 11 wavelengths in common with the original set. The visible identity signal is therefore robust, but the exact reduced-band specification should be interpreted as exploratory rather than definitive.

### 3.2. PCA as a Mechanistic Bridge, Not a Compression Trick

PCA was used here as an interpretation tool rather than a compression step for model fitting. The first three components captured 97.42% of the total variance across the 563-band spectra, indicating that the dominant spectral organisation is low-dimensional even though the raw signatures are high-dimensional. The broad, contiguous loading structures imply latent drivers such as global albedo, pigment-related visible absorption and longer-wave contrast, rather than isolated single-band artefacts. In score space, accessions occupied shifted but partially overlapping regions, which is exactly the geometry expected to generate concentrated neighbour-to-neighbour errors rather than uniform confusion.

The score-space geometry adds a second mechanistic layer. The ROI-level spectra formed a continuous manifold with accession-specific shifts and a partial overlap, implying heterogeneous separability [13,16,23,25]. For example, some accessions occupy extremes of this low-dimensional organisation, whereas many are concentrated in a dense central region. This predicts structured errors downstream: misclassification should concentrate among “spectral neighbours” rather than diffuse uniformly across labels because some accessions genuinely occupy overlapping regions of optical phenotype space under the acquisition conditions used. The same logic helps explain Figure S1: a high pairwise Pearson correlation among accession means, even for some visually distinct materials, does not preclude accurate classification because the correlation summarises similarity only two profiles at a time, whereas the trained models use multivariate decision boundaries defined by the joint behaviour of hundreds of wavelengths.

Because PCA is unsupervised, large absolute loadings indicate wavelength regions that drive overall variance, not necessarily class discrimination; therefore, we complement PCA loadings with supervised/association-based wavelength attribution (one-versus-rest R^2^ mapping and ReliefF).

### 3.3. Band Relevance, Sensor Design, and Meaning of “Compact” Discrimination

Reduced-band analysis shows both the promise and the limitation of compact sensor design. The importance of ReliefF was concentrated in the visible range, and the global permutation importance plus SHAP attribution peaked at approximately 577–598 nm, with 597.54 nm emerging as the strongest global wavelength in the permutation analysis and 597.54 nm as the strongest mean absolute SHAP wavelength. These windows are therefore strong candidates for future multispectral prototypes. This interpretation is consistent with recent wavelength selection studies showing that compact panels can be informative when selection is supervised and redundancy is handled explicitly, although the stability of the final panel remains criterion- and context-dependent [41,42,43,44].

However, compact is not equivalent to complete. In the original ReliefF-selected 16-band benchmark, the best model reached 68.25% macro-F1 and 68.54% balanced accuracy, but five of those bands were at the spectral edges. After edge-band trimming, the alternative 16-band configuration decreased to 60.67% and 60.94%, whereas the full-spectrum benchmark reached 96.35% and 96.35% for the same metrics, and the 555-band edge-trimmed representation remained essentially unchanged (96.13%/96.15%). In other words, the selected visible windows carry real identity signals, but a full spectral context is still needed to resolve the most confusable accessions under controlled laboratory conditions. Global SHAP attribution and the model-specific permutation/SHAP profiles that support this finding are reported in Figures S6 and S12–S19.

### 3.4. Why Do Linear Models Reach Near-Ceiling Accuracy, and What Residual Confusion Is Revealed?

Within the classical suite, the full-spectrum benchmark was dominated by linear discriminant analysis and linear SVM, indicating that much of the accession structure becomes close to linearly separable once the full 563-band reflectance profile is retained. Across the reduced-band benchmarks, the subspace discriminant was the strongest classifier, but the performance fell well below the full-spectrum setting—especially after edge-band trimming. This contrast suggests that a broad spectral context contributes more to class boundary stability than a compact top-k ranking alone can preserve.

The resulting performance level is comparable to the near-ceiling accuracies reported for seed and grain accession identification under tightly controlled settings, particularly when the number of classes is moderate and the acquisition conditions are stable [21,34,45,46,47]. Here, the substantive contribution is therefore not a single headline score but a traceable benchmark that links wavelength attribution (association maps and ReliefF), a low-dimensional structure (PCA loadings and score geometry) and the residual error structure across model families. This provides quantitative guidance for reduced-band sensor design and for full-spectrum classifier development under controlled conditions. Comparable seed-classification performance has also been reported for rice, soybean and aged seed workflows, although the exact architecture ranking still shifts with respect to the crop, wavelength range and acquisition protocol [20,30,34,35,48,49,50].

### 3.5. Deep Learning: Why Does the Simplest Architecture Win?

The deep learning comparison points in the same direction. The best architecture was not the most complex, but MLP_Wide, which reached 84.90% accuracy and 84.47% macro-F1 on the independent test set. CNN1D_Deep and TCN1D formed a secondary tier, whereas the recurrent and attention-based models underperformed more markedly. Under a moderate-data regime with smooth one-dimensional signatures, simpler feed-forward inductive biases appear better matched to the signal-to-sample ratio than heavier sequence models do. Even so, the best deep model clearly remained below the strongest full-spectrum classical learners.

This finding is also useful from translational and benchmarking perspectives [27,29,51]. For example, our second hypothesis suggests that if a compact architecture already performs well, it provides a robust baseline for future deployment-oriented studies and for subsequent interpretability analyses, aligning with the broader push in agricultural AI for models that are not only accurate but also stable and auditable. This is consistent with the use of hyperspectral imaging sensors with narrow spectral bandwidths that capture the finest material nuances [13,52,53]. In this respect, a multispectral sensor with narrow, deliberately placed bands spanning a wide spectral range may ultimately be preferable to full hyperspectral cameras when throughput, cost and robustness dominate, although engineering choice still requires explicit external validation. The equivalence between accuracy and macroaveraged recall suggests that the dataset was well balanced and that the models exhibited a symmetric error pattern across classes, with similar true-positive rates for each class. As a result, the overall proportion of correct predictions mirrored the average recall, leading both metrics to converge (Table 1 and Table 2).

### 3.6. Deployability: Accuracy Is Not the Only Axis That Matters

Coupling predictive performance to throughput, training time and model size remains essential for deployment because models with similar scores can differ by orders of magnitude in computational cost. In the reduced-band benchmark, the throughput ranged from 1.55 × 10^4^ to 9.90 × 10^6^ observations s^−1^, the training time ranged from 9.44 × 10^−4^ to 2.37 s, and the model size ranged from 4.39 kB to 8.31 MB. These contrasts matter for embedded systems, conveyor sorting and real-time inspection.

The recent hyperspectral learning literature shows that deep architectures can perform strongly, but their gains depend on sample size, domain shift and the geometry of the signal itself. Our benchmark fits a regime in which simpler models remain highly competitive: the spectra are smooth, the low-dimensional structure is strong, and acquisition conditions are tightly controlled [54]. Transfer across sessions, instruments and illumination settings therefore remains a central unresolved barrier, and in-lab performance should not be extrapolated to operational deployment without explicit domain-shift validation. This unresolved transfer problem is reflected in the rapid growth of hyperspectral domain-adaptation and cross-scene transfer methods, which are increasingly discussed alongside explainable-AI constraints for operational remote sensing [54,55,56,57,58].

Taken together, the results support a mechanistic and deployment-aware view of hyperspectral seed analytics. Accession identity is recoverable with high fidelity from the full reflectance profile, but the strongest reduced-band windows sit in the visible range and therefore provide actionable guidance for future multispectral designs. The combination of PCA, one-versus-rest association mapping, ReliefF, permutation importance and SHAP helps explain both why performance is high in the full-spectrum setting and why aggressive compression degrades the classifier ranking.

Although the NIR–SWIR reflectance encodes absorptions associated with major chemical bonds (e.g., O–H, C–H, and N–H overtones/combination bands), relatively few top-ranked wavelengths were assigned to the SWIR region in the present attribution analyses [29,45,55,56]. This does not imply that the SWIR is irrelevant; rather, under the controlled laboratory conditions used here, accession discrimination appears to be dominated by the seed coat colour, albedo and surface optical phenotype, whereas the SWIR contributes more to the complementary full-spectrum context than a compact set of individually dominant bands do. Because we did not measure the biochemical composition in this classification benchmark, we cannot determine whether the limited SWIR attribution reflects genuinely weaker compositional contrasts among accessions or simply whether such contrasts were secondary to colour-driven discrimination in this dataset [45]. We therefore avoided assigning specific absorptions to specific constituents [57,58]. Composition-focused modelling is treated separately and/or is a priority for future multilot validation.

From an implementation perspective, the proposed workflow should be read as a deployment-oriented hypothesis rather than as a validated operational seed-sorting solution [45,56]. In principle, a line-scan (push-broom) hyperspectral sensor—or a reduced-band multispectral design guided by the highlighted visible windows—could be mounted above a conveyor belt, with automated seed segmentation generating per-seed masks/ROIs and extracting one spectrum per seed on the fly. However, any such translation would first require automated segmentation, illumination control, cross-session testing and explicit domain-shift validation before compact models such as linear discriminant analysis could be considered reliable outside the present controlled laboratory setting [56,57,58].

Preprocessing sensitivity analysis revealed that Savitzky–Golay smoothing with clipping was a stable default and that optional baseline correction or SNV influenced interpretability more strongly than the identity of the strongest full-spectrum classifiers did. Nevertheless, preprocessing must be fixed before external validation so that performance differences are not conflated with analytical drift [56,59].

Although these results demonstrate strong discrimination under controlled conditions, performance under non-ideal acquisition (illumination shifts, partial occlusion/stacking and surface contamination) was not quantified here and remains a priority for future deployment-oriented validation.

### 3.7. Limitations and Deployment-Oriented Validation

This study was designed as a controlled, traceable benchmark to establish an internal validity baseline for accession discrimination, wavelength attribution and fair model comparison. Several deployment-relevant factors were not quantified, including illumination variability, seed stacking or partial occlusion, surface contamination, cross-sectional drift and cross-instrument transfer [59,60]. Future validation should therefore include deliberate stress tests together with explicit domain-shift experiments.

ROI spectra were extracted from manually delineated regions following a fixed rule-set to reduce subjectivity. Nevertheless, robust operational deployment requires automated segmentation and ROI extraction [45,61,62,63]. A practical next step is to integrate a lightweight thresholding- or contour-based masker or a trained detector/segmenter and to verify that automated ROIs preserve both spectral fidelity and classification performance relative to manual ROIs under the same seed-level split protocol.

In addition, variability across production lots (e.g., moisture content, storage age, and associated changes in seed coat glossiness/roughness) may shift reflectance signatures and should be explicitly quantified in future multilot validation to assess model transferability. The prediction of physical and chemical seed properties from spectral data is outside the scope of the present classification benchmark and is addressed in a companion study currently under review. Seed lot history, including storage and pretreatment, can also modify physical or biochemical properties relevant to downstream sensing workflows and should therefore be controlled explicitly during external validation [19,20,64,65,66].

Finally, the reduced-band ranking was generated on the full preprocessed dataset and subsequently used to define exploratory sensitivity analyses. The resulting 16-band benchmark is therefore informative for wavelength attribution and sensor design, but it should not be interpreted as a fully nested, leakage-free feature selection experiment. Claims about field-scale robustness or definitive multispectral sensor specifications should remain provisional until external domain-shift validation is completed.

## 4. Materials and Methods

### 4.1. Plant Material, Genetic Background and Experimental Design

Thirty-two grain–legume accessions were analysed, comprising thirty traditional common bean (*Phaseolus vulgaris* L.) landrace cultivars and two non-*Phaseolus* grain–legumes that are locally classified as ‘Feijões’ (A27: *Vigna angularis* (Willd.) Ohwi & Ohashi; A28: *Cajanus cajan* (L.) Huth; Table S1). All materials were sourced from a maintained germplasm bank, with curated accession identities and documented provenances. The use of curated germplasm accessions provides a realistic test of authentication at the interface of conservation, breeding and seed quality control.

The selected landraces represent a broad spectrum of traditional bean types in Brazil, encompassing substantial phenotypic, biochemical and genetic diversity preserved through long-term farmer selection and regional adaptation processes. The collection comprised the following cultivars: (1) Feijão Amendoim Roxo, (2) Feijão Carioca, (3) Feijão Preto Gigante, (4) Feijão Branco Manteiga, (5) Feijão Rosinha, (6) Feijão Branco Gigante, (7) Feijão Canário, (8) Feijão Preto Mocotó, (9) Feijão Verde, (10) Feijão Vermelho Bolinha, (11) Feijão Branco Bolinha, (12) Feijão Mouro Gigante, (13) Feijão Roxinho, (14) Feijão Creme, (15) Feijão Vermelho Gigante, (16) Feijão Jalo, (17) Feijão Amendoim Bege, (18) Feijão Boreal Rosa, (19) Feijão Chocolate, (20) Feijão Amendoim Rosa, (21) Feijão Olho de Pombo, (22) Feijão Bico de Ouro, (23) Feijão Amendoim, (24) Feijão Ovo de Tito-Tico, (25) Feijão Amendoim Verde, (26) Feijão Rajado, (27) Feijão Azaki, (28) Feijão Guandu, (29) Feijão Mouro, (30) Feijão Mancá, (31) Feijão Vermelho and (32) Feijão Rapa Cuiua (Table S1).

Accession information and prior characterisation records from the germplasm bank indicate that the collection spans substantial phenotypic diversity across seed coat colour, patterning and grain morphology, supporting its suitability as a challenging benchmark for spectral discrimination under controlled laboratory conditions.

### 4.2. Hyperspectral Image Acquisition and Spectral Consistency Analysis

Hyperspectral image acquisition was conducted with an Aisa-FENIX hyperspectral sensor imaging system (Specim, Oulu, Northern Ostrobothnia, Finland) mounted on a laboratory push-broom scanning platform. The instrument provides nominal VIS–NIR–SWIR coverage through the VNIR and SWIR channels; after calibration and export in the present workflow, the analysed wavelength axis comprised 563 bands spanning 449.54–2399.17 nm. The acquisition line rate was 12 frames s^−1^.

Three scanning sessions were performed, each comprising multiple Petri dishes, yielding a total of *n* = 3200 seeds. For each scan, approximately 50 seeds were arranged in a single layer on a stable, non-reflective background to minimise overlap and facilitate manual region-of-interest delineation. The same dishes used for complementary point-based spectroradiometry were scanned when applicable, allowing qualitative comparison between point spectra and image-derived reflectance curves.

Image acquisition was performed at a fixed sensor–sample distance of 85 cm to standardise spatial sampling and minimise bidirectional reflectance distribution function (BRDF) effects. Measurements were conducted in a controlled laboratory environment, with reflectance referenced to a Spectralon^®^ diffuse reflectance standard (Labsphere Inc., Northern Sutton, NH, USA), characterised by near-Lambertian behaviour across the VIS–NIR–SWIR range, supporting robust and reproducible calibration. Acquisition was performed under controlled laboratory conditions; twelve 20 W halogen lamps were evenly placed around the scan window to provide uniform illumination, the ambient temperature was maintained at 25 °C, and external light interference was eliminated. The camera’s internal cooling was activated, and the camera was allowed to stabilise for 30 min before the acquisition of spectral data. Radiometric referencing and dark current correction followed standard procedures, and the reflectance was computed relative to a calibrated white reference.

#### 4.2.1. Radiometric Calibration and Reflectance Conversion

Radiometric calibration was carried out via CaliGeoPRO^®^ software, version 2.2 (Specim, Oulu, Northern Ostrobothnia, Finland). The calibration workflow incorporated raw hyperspectral data files generated by the Aisa-FENIX sensor (.raw), together with sensor-specific radiometric calibration files (.cal and .LUT) and dark reference headers (.hdr). This procedure corrected for sensor noise, dark current and system-specific radiometric response.

The radiance-calibrated outputs were subsequently converted to reflectance units via ENVI Classic^®^ software version 5.4 (Harris Geospatial Solutions, Boulder, CO, USA). A manufacturer-validated algorithm tailored to the Aisa-FENIX data was applied through the scan normalisation module. The radiance data were combined with dark reference measurements, and the reflectance was computed via the option “Reflectance using a subset of the image as a white reference”, with the Spectralon^®^ panel region explicitly defined as the reference target. This process yielded reflectance-calibrated hyperspectral images (radiance_refl.dat).

#### 4.2.2. Region of Interest Extraction and Spectral Signature Generation

After reflectance calibration, the hyperspectral data cubes were exported to ENVI Classic^®^ v5.4 (Harris Geospatial Solutions, Boulder, CO, USA) for subsequent processing. For each seed, one region of interest (ROI) was manually defined over a central representative area while excluding marginal regions in which curvature, background mixing or specular artefacts could distort reflectance. The ROI was defined as a 10 × 10 pixel square window (100 pixels). Within this window, 10 representative interior pixels were selected after excluding obvious highlights, shadows and visible contaminants, and their mean reflectance was computed at each wavelength to yield one spectrum per seed.

In total, this workflow produced *n* = 3200 seed-level spectral signatures linked to accession labels A01–A32 (100 spectra per accession). ROI delineation followed a fixed rule-set to minimise subjective variability: (1) the ROI window was placed within the central seed body to avoid boundary curvature and background mixing; (2) pixels affected by specular highlights, shadows, or visible contaminants were excluded; (3) a fixed ROI window size of 10 × 10 pixels was used as a standardised sampling frame for all seeds whenever feasible; and (4) within this window, 10 representative interior pixels were selected and averaged to obtain exactly one mean spectrum per seed. A small placement sensitivity check (three alternative central placements on a subset of seeds) indicated negligible changes in the resulting mean spectra relative to between-accession differences, supporting the use of a single spectrum per seed in the main benchmark.

The analysed wavelength axis used throughout this manuscript comprised 563 bands spanning 449.54–2399.17 nm. In an additional quality control analysis, spectral-edge trimming removed eight bands within 10 nm of the lower and upper limits, yielding a 555-band representation spanning 459.68–2388.25 nm. All primary full-spectrum analyses reported here use the 563-band representation unless stated otherwise.

### 4.3. Organisation of the Spectral Dataset and Python Implementation

All post-extraction processing was carried out using Python version 3.13.9 (Python Software Foundation, Wilmington, DE, USA) in a single, fully scripted pipeline to maximise transparency and reproducibility. The calibrated reflectance measurements were arranged into a feature matrix X ∈ ℝ^(3200 × 563), where rows correspond to seed-level ROI spectra and columns correspond to wavelengths. Each spectrum was paired with an accession identifier (A01–A32), and wavelength positions were stored in a companion vector λ expressed in nm.

### 4.4. Spectral Preprocessing

Each extracted ROI was represented by a single reflectance vector comprising P = 563 bands spanning 449.54–2399.17 nm. Accessions were indexed via codes A01–A32, corresponding directly to the cultivar numbering defined in Section 4.1 and reported in Table S1.

Spectral preprocessing followed the scripted workflow used to generate the reported outputs. Polynomial baseline correction (degree 2) and standard normal variate (SNV) normalisation were implemented but disabled in the primary analyses. The main reported workflow used Savitzky–Golay smoothing (window length = 11, polynomial order = 2) followed by clipping to [0, 1] to retain physically plausible reflectance values. The smoothing step follows the original Savitzky–Golay formulation [67].

When the SNV was used for sensitivity analysis, each spectrum was centred on its own mean and scaled by its own standard deviation so that the relative shape rather than absolute reflectance dominated the transformed signal. Because SNV changes the physical scale of reflectance, it was treated as a sensitivity analysis option rather than as the default representation used for the main benchmark.

### 4.5. Descriptive and Integrative Spectral Analyses

Exploratory spectral analyses included accession-wise mean spectra, PERMANOVA on the Euclidean distance matrix of the preprocessed spectra (999 permutations), one-versus-rest Pearson correlation profiles across wavelengths, coefficient-of-determination mapping (R^2^ = r^2^), and hierarchical clustering of accession-mean spectra using Euclidean distance. These analyses were used to characterise the global variance structure, local wavelength associations and between-accession spectral neighbourhoods.

For one-versus-rest association mapping, the accession labels were encoded as binary indicators (1 = focal accession; 0 = all remaining accessions); thus, the resulting wavelength-wise correlations quantified the class-specific association strength without imposing any ordinal structure on the accession IDs.

For presentation, correlation maps retained the sign of r, whereas attribution strength was summarised as the coefficient of determination (R^2^). This dual representation allowed the direction of association and its magnitude to be visualised separately across the spectrum. Hierarchical clustering was applied to the accession-mean spectra using Euclidean distance and average linkage to describe spectral similarity at the accession level. Representative seed images were then arranged in dendrogram leaf order to facilitate visual comparison between spectral neighbourhoods and seed coat phenotypes.

### 4.6. Principal Component Analysis

Prior to PCA, each wavelength band was standardised to zero mean and unit variance across seeds so that broad-intensity regions did not dominate the decomposition.

PCA was then applied to the standardised matrix to obtain score and loading representations for both individual spectra and accession centroids.

The explained variance ratios were exported for the first 20 components, and both individual-spectrum and cultivar-centroid score plots were generated. Loading vectors (PC1–3) were analysed to identify the wavelength regions contributing most strongly to principal component separation.

To assess whether the spectral profiles differed among the accessions across the full wavelength space, we performed permutational multivariate analysis of variance (PERMANOVA) on the Euclidean distance matrix computed from the preprocessed ROI spectra, with accessions used as the grouping factor and 999 permutations. The resulting F test and permutation *p*-values are reported in the hyperspectral curves.

### 4.7. Feature Ranking and Selection via Relief

Feature ranking was used to quantify which wavelengths contributed most to the separability of the accessions and to define an exploratory reduced-band representation for sensitivity analysis and future sensor design considerations.

$$w_{j}\leftarrow w_{j}-\frac{1}{mk}\sum_{h\in H_{i}}\left|x_{ij}-x_{hj}\right|+\frac{1}{mk}\sum_{m\in M_{i}}\left|x_{ij}-x_{mj}\right|$$

where *wj* is the ReliefF weight for wavelength band j, m is the number of sampled reference spectra (iterations), k is the number of nearest neighbours, *Hi* is the set of k nearest hits (same accession as spectrum i), *Mi* is the set of k nearest misses (different accessions), and *xsj* denotes the reflectance of spectrum s at band j (s = i, h, r).

An approximate ReliefF implementation was applied to the preprocessed spectra. In each iteration, a sampled spectrum was compared with its nearest hit and nearest miss, and per-band weights were updated by penalising within-class absolute differences and rewarding between-class separation. The resulting rank order was used for interpretation and for the exploratory 16-band sensitivity benchmark; it was not used as a nested feature selection step inside each training fold.

#### 4.7.1. ReliefF Implementation Details and Hyperparameters

ReliefF weighting was implemented in Python, using NumPy version 2.2.6 (NumPy Developers, Austin, TX, USA) for the weight updates and scikit-learn’s NearestNeighbors for neighbour retrieval. The spectra were internally min–max scaled to [0, 1] for the neighbour searches, and the Euclidean (L2) distance was used throughout. The neighbour-search implementation and associated utilities followed the scikit-learn framework [67].

The neighbourhood size was 20 and 800 randomly sampled reference spectra were evaluated (seed = 42). For each reference spectrum, the nearest same-class and different-class neighbours were identified after excluding the query spectrum itself. Because the ranking served wavelength attribution and exploratory sensitivity analysis, it was computed on the full preprocessed dataset rather than on a training-only subset; the reduced-band results should therefore be interpreted as descriptive/exploratory rather than as a fully nested leakage-free feature selection benchmark. 

### 4.8. Classification Modelling Framework

Accession identification was framed as a 32-class classification problem. For the classical benchmark and spectral representation sensitivity analyses, a fixed stratified seed-level partition was used: 70 seeds per accession (*n* = 2240) formed the development subset, and 30 seeds per accession (*n* = 960) formed the fully independent test subset. Within the classical benchmark, the development subset was divided evenly into training and validation subsets (35 seeds per accession each; 35:35:30 overall), and the same split indices were reused across all the classical models and spectral representations. Each seed contributed a single ROI-averaged spectrum, preventing any pixel- or seed-level overlap between the development and independent test data.

The predicted labels on the independent test set were used to compute the overall accuracy and error rate as follows:

$$\mathrm{Accuracy}=\frac{1}{N}\sum_{i=1}^{N}oI\left(\hat{y_{i}}=y_{i}\right)$$

Error rate=1−Accuracy

$$C_{ab}=\sum_{i=1}^{N}oI\left(y_{i}=a\right)I\left(\hat{y_{i}}=b\right)$$

Confusion matrices were computed on the independent test set and optionally row-normalised for visual comparison of the per-class error structure. The precision, recall and F1 score were summarised as macro, micro- and support-weighted averages. Because the independent test set was perfectly balanced (30 seeds per accession), the weighted and macro summaries were numerically very similar. Training time, prediction throughput, total misclassification cost and serialised model size were also recorded to characterise computational feasibility.

$${\mathrm{Precision}}_{c}=\frac{TP_{c}}{TP_{c}+FP_{c}}$$

$${\mathrm{Recall}}_{c}=\frac{TP_{c}}{TP_{c}+FN_{c}}$$

$$F1_{c}=\frac{2\;{\mathrm{Precision}}_{c}\;{\mathrm{Recall}}_{c}}{{\mathrm{Precision}}_{c}+{\mathrm{Recall}}_{c}}$$

$${\mathrm{Precision}}_{w}=\sum_{c}\frac{n_{c}}{N}\;{\mathrm{Precision}}_{c}$$

$${\mathrm{Recall}}_{w}=\sum_{c}\frac{n_{c}}{N}\;{\mathrm{Recall}}_{c}$$

$$F1_{w}=\sum_{c}\frac{n_{c}}{N}\;F1_{c}$$

For the baseline benchmark, we additionally report a composite coefficient defined as the arithmetic mean of the three support-weighted metrics (precision, recall and F1 score): C = (precision + recall + F1 score)/3.

### 4.9. Model Optimisation Curves and Probabilistic Performance Summaries

To replicate optimisation-style diagnostics, two model families were further analysed via simplified grid evaluations using only the training data and an internal validation split. For k-nearest neighbours, the number of neighbours was varied over a finite range constrained by the dataset size, and the validation classification error was recorded to identify an appropriate k. For multilayer perceptron classification, a discrete set of hidden-layer configurations was evaluated under the same protocol to identify a performant architecture under fixed optimisation settings. The independent test set was not used for model selection.

When classifiers provide probabilistic outputs via predict_proba or continuous decision scores via a decision function, score matrices on the independent test set are used to compute microaveraged receiver operating characteristic (ROC) and precision–recall (PR) curves by binarizing class labels in a one-versus-rest manner and pooling across all classes. In addition, micro- and macroaveraged ROC-AUC and average precision summaries were retained for tabular reporting. For binary scoring of a positive class, the ROC definitions were as follows:

$$\mathrm{TPR}=\frac{\mathrm{TP}}{\mathrm{TP}+\mathrm{FN}}$$

$$\mathrm{FPR}=\frac{\mathrm{FP}}{\mathrm{FP}+\mathrm{TN}}$$

$$\mathrm{Precision}=\frac{\mathrm{TP}}{\mathrm{TP}+\mathrm{FP}}$$

$$\mathrm{Recall}=\frac{\mathrm{TP}}{\mathrm{TP}+\mathrm{FN}}$$

The area under the ROC curve (AUC) and average precision (AP) were computed as scalar summaries of the ranking quality under both the pooled microaverage and class-averaged macro formulations.

### 4.10. Deep Learning Models on 1D Spectral Signatures

Deep learning was implemented directly on the one-dimensional reflectance vectors, treating each spectrum as a fixed-length sequence of length P = 563. The same fixed 70:30 seed-level partition was used to define the independent test set (30 seeds per accession; *n* = 960). Within the 70% development portion, 15% was reserved for validation during training, leaving *n* = 1904 spectra for parameter optimisation and *n* = 336 for model selection and early stopping.

The evaluated architectures included three multilayer perceptrons (MLP_1D, MLP_Wide and MLP_Deep), two one-dimensional convolutional neural networks (CNN1D and CNN1D_Deep), ResNet1D, Inception1D, TCN1D, BiLSTM1D and Transformer1D. All the models were trained with the Adam optimiser (learning rate = 1 × 10^−3^), a batch size = 32 and a maximum of 25 epochs; early stopping (patience = 6) restored the weights associated with the lowest validation loss.

For a sample i with a true class label $y_{i}$ and a predicted class probability $p_{i,c}$, the multiclass cross-entropy loss is

$$L=-\frac{1}{N}\sum_{i=1}^{N}log\;\left(p_{i,y_{i}}\right)$$

The deep learning performance on the independent test set was summarised in terms of accuracy, macroaveraged precision, macroaveraged recall and macroaveraged F1 score, together with the number of trainable parameters as a measure of model capacity. Training and validation learning curves were retained for each architecture to document optimisation dynamics and to diagnose overfitting under the shared training budget.

### 4.11. Reproducibility and Figure Generation

All figures, tables and intermediate outputs reported in this work, including mean spectra plots, heatmaps, PCA scores and loadings, dendrograms, feature importance profiles, model comparison panels, confusion matrices and deep learning training curves, were generated programmatically from the same Python version 3.13.9 script pipeline used for model training and evaluation. Fixed random seeds were used across data splitting and model initialisation steps wherever supported (Figure 2). Model evaluation and all reported metrics were computed on the independent held-out test set described above, ensuring that the reported performance reflects generalisation beyond the fitted training data [67].

All analyses and figure generation were performed in Python version 3.13.9 (Python Software Foundation, Wilmington, DE, USA) via NumPy version 2.2.6 (NumPy Developers, USA), Pandas version 2.3.3 (Pandas Development Team, Austin, TX, USA), Matplotlib version 3.10.5 (Matplotlib Developers, New York, NY, USA), Seaborn version 0.13.2 (Seaborn Developers, New York, NY, USA), SciPy version 1.16.3 (SciPy Developers, Austin, TX, USA), scikit-learn version 1.7.2 (Scikit-learn Developers, New York, NY, USA) [68] and openpyxl version 3.1.5 (Openpyxl Developers, Cambridge, MA, USA). The deep learning models were implemented in PyTorch version 2.2.2 (PyTorch Developers, Menlo Park, CA, USA). All random operations (data split, model initialisation, and bootstrap resampling) use fixed random seeds to ensure reproducibility.

**Figure 9:**
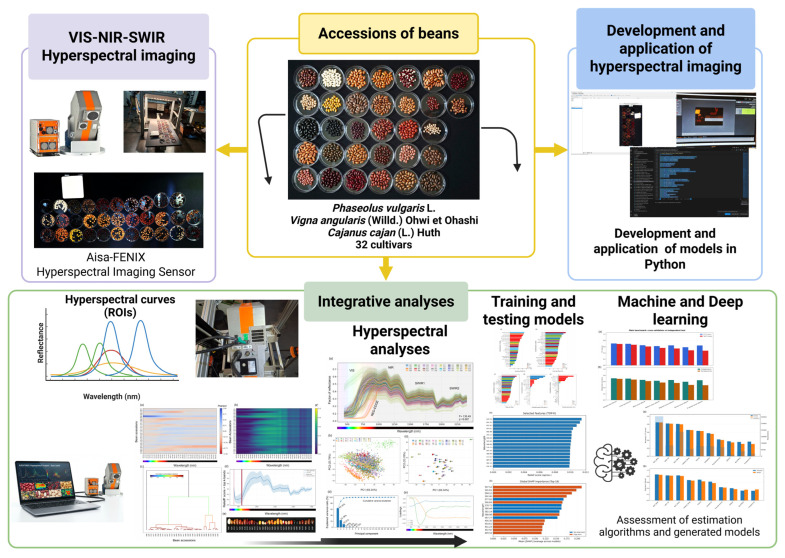
Overview of the experimental and analytical workflow. Seeds from 32 bean accessions/cultivars (including *Phaseolus vulgaris* L., *Vigna angularis* (Willd.) Ohwi & Ohashi, and *Cajanus cajan* (L.) Huth) were imaged using VIS–NIR–SWIR hyperspectral imaging (Aisa-FENIX). Regions of interest (ROIs) were used to extract the seed-level reflectance spectra, which were then integrated into the hyperspectral preprocessing and analysis. The processed spectra supported Python-based development and evaluation of conventional machine learning models and deep learning approaches, followed by performance assessment of the generated models.

Model development and computational routines were executed on a workstation equipped with an NVIDIA GeForce RTX 4090 GPU (NVIDIA Corporation, Santa Clara, CA, USA), an Intel Core i9-14900HX CPU (Intel Corporation, Santa Clara, CA, USA), 128 GB RAM (Corsair Vengeance DDR5 6400 MHz, Corsair Inc., Fremont, CA, USA) and NVMe storage (Movespeed 4TB SSD, M.2 2280, Movespeed, Shenzhen, China), supporting efficient handling of high-dimensional spectral datasets and enabling consistent benchmarking across classical and deep learning approaches (Figure 9).

## 5. Conclusions

Full-range VIS–NIR–SWIR hyperspectral imaging of dry seeds generated stable accession-specific signatures that supported non-destructive identification across 32 grain–legume accessions. Across the 563 analysed bands (449.54–2399.17 nm), the first three principal components captured 97.42% of the total variance, and wavelength attribution concentrated the strongest reduced-band signal within two visible windows centred at 449.54–456.30 nm and 577.02–597.54 nm. Full-spectrum benchmarking revealed that linear discriminant analysis reached 96.35% macro-F1 and 96.35% balanced accuracy. In the original ReliefF-selected 16-band benchmark, the subspace discriminant reached 68.25% macro-F1 and 68.54% balanced accuracy; after edge-band trimming, the alternative 16-band configuration decreased to 60.67% and 60.94%, respectively. In the deep learning comparison, MLP_Wide was the strongest one-dimensional architecture (84.90% accuracy; 84.47% macro-F1), outperforming the convolutional, recurrent and attention-based alternatives but remaining below the strongest full-spectrum classical models. Together, these results establish a controlled benchmark for accession-level seed authentication under laboratory conditions, show that the most compact discriminatory signal was concentrated in the visible range, and indicate that the SWIR contributed mainly to the complementary full-spectrum context rather than through many individually dominant bands. Wavelength-ranking analyses therefore provide mechanistic guidance for future multispectral sensor design, whereas the reduced-band results should be interpreted as exploratory because the ranking step was not nested inside model training. However, neither operational deployment nor biochemical attribution should be inferred beyond the present controlled benchmark until explicit multilot, cross-session, and composition-linked validation has been performed.

## Figures and Tables

**Table 1:** Sensitivity summary across spectral representations. For each representation, the table reports the wavelength coverage retained after preprocessing, the number of bands used, the best-performing model, and the corresponding macro-F1 and balanced accuracy on the held-out test set. The final 16-band row corresponds to the edge-trimmed alternative configuration.

Representation	Wavelength Coverage (nm)	Bands	Best Model	Macro-F1 (%)	Balanced Accuracy (%)
Full spectrum	449.54–2399.17	563	Linear discriminant	96.35	96.35
Trimmed spectrum	459.68–2388.25	555	Linear discriminant	96.13	96.15
Trimmed spectrum + top-k	575.31–600.96	16	Subspace discriminant	60.67	60.94

**Table 2:** Top-performing models in the full-spectrum sensitivity benchmark, ranked by held-out macro-F1 score. The classifier family, macro-F1 score and balanced accuracy are reported for the eight strongest models.

Rank	Model	Group	Macro-F1 (%)	Balanced Accuracy (%)
1	Linear discriminant	Discriminant	96.35	96.35
2	Linear SVM	SVM	94.17	94.17
3	Medium neural network	Neural Network	91.57	91.56
4	Narrow neural network	Neural Network	91.10	91.15
5	Wide neural network	Neural Network	88.44	88.54
6	Bi-layered neural network	Neural Network	87.73	87.71
7	Subspace discriminant	Ensemble	84.75	85.00
8	Tri-layered neural network	Neural Network	81.66	81.98

**Table 3:** Independent test set performance of the one-dimensional deep learning architectures evaluated for grain–legume seed accession classification using hyperspectral reflectance curves. The accuracy, F1 score, precision and recall are reported as percentages; the parameters indicate the total number of trainable model parameters, and the number of epochs indicates the number of training epochs completed.

Model	Accuracy (%)	F1 Score (%)	Precision (%)	Recall (%)	Parameters	Epochs
MLP_Wide	84.90	84.47	86.27	84.90	305,184	25
MLP_1D	81.98	81.38	82.96	81.98	82,528	25
MLP_Deep	80.94	79.84	82.85	80.94	187,616	25
CNN1D_Deep	65.73	65.29	67.49	65.73	57,536	25
TCN1D	63.23	62.83	68.89	63.23	77,344	25
ResNet1D	57.60	55.91	61.99	57.60	77,600	25
Transformer1D	42.08	40.66	43.76	42.08	69,152	25
CNN1D	37.40	34.35	40.66	37.40	12,832	25
Inception1D	36.46	30.71	31.92	36.46	19,504	22
BiLSTM1D	35.73	31.01	32.51	35.73	38,432	21

## Data Availability

The original contributions presented in this study are included in the article/Supplementary Material. Further inquiries can be directed to the corresponding author.

## Supplementary Materials

The following supporting information can be downloaded at: https://www.mdpi.com/article/10.3390/plants15060933/s1, Figure S1. Correlation matrix of the accession-mean spectra across A01–A32, showing pairwise Pearson correlation coefficients among the preprocessed class means. The plot summarises the spectral similarity across accessions and highlights both closely related and more distinct spectral neighbourhoods within the dataset. Figure S2. Supervised wavelength ranking based on ANOVA eta^2^ and ReliefF, with the selected wavelengths indicated on both profiles to compare the global between-class effect size and supervised local feature relevance. Figure S3. Spectral quality control and edge-band trimming. Panels show the mean signal, coefficient of variation, local roughness, and placement of selected wavelengths over the trimmed mean spectrum, providing the rationale for removing unstable spectral-edge regions. Figure S4. Agreement between cross-validation and independent test performance in the main benchmark. (a) Macro-F1 score and (b) balanced accuracy for the best-performing classical models, highlighting the generalisation gap between development and held-out evaluation. Figure S5. Internal optimisation diagnostics and ranking-quality summaries for representative baseline workflows. Panels show validation-error trajectories during model tuning and the corresponding ROC and precision–recall curves used to summarise ranking performance. Figure S6. Top-k wavelength selection and global SHAP attribution for the reduced-band configuration. (a) Relief-based ranking of the selected wavelengths. (b) Mean absolute SHAP importance across models, contrasting edge bands and interior bands. Figure S7. (a) Accuracy and (b) macro-F1 score of the additional baseline classifiers evaluated on the independent test set. Figure S8. Row-normalised confusion matrices for the 12 highest-ranking classifiers in the benchmark, ordered from best to worst overall accuracy. The percentages are shown in cells to facilitate comparisons of class-specific recall and dominant confusion patterns. Figure S9. Row-normalised confusion matrices for classifiers ranked 13th to 25th, and the benchmark ordering is shown in Figure S8. Percentages are displayed in cells to reveal how misclassification patterns change in the lower-performing models. Figure S10. Microaveraged one-versus-rest ROC and precision–recall curves for the main benchmark models, summarising the ranking performance across all classes. Figure S11. Heatmap summary of model sensitivity across spectral variants and comparison of the best score obtained within each variant. (a) The heatmap shows the macro-F1 test results by model and spectral representation, whereas panel (b) shows the top-performing model for each variant. Figure S12. Model-specific permutation-importance profiles for the global reference and the models with the closest agreement with the global wavelength ranking (panel set 1 of 4). The bars represent the importance of macro-F1-based permutations across the selected wavelengths. Figure S13. Continuation of model-specific permutation-importance profiles for models with high concordance with the global wavelength ranking (panel set 2 of 4). Figure S14. Continuation of model-specific permutation-importance profiles for models with intermediate concordance and hybrid wavelength-importance profiles (panel set 3 of 4). Figure S15. Continuation of model-specific permutation-importance profiles for models with the most divergent, sparse, or contrasting wavelength-importance patterns (panel set 4 of 4). Figure S16. Model-specific SHAP profiles for the models most aligned with the global importance pattern (panel set 1 of 4). The bars represent the mean absolute SHAP values across the selected wavelengths. Figure S17. The continuation of model-specific SHAP profiles for models with high, although not identical, agreement with the global importance pattern (panel set 2 of 4). Figure S18. Continuation of model-specific SHAP profiles for models with intermediate selectivity and partially divergent attribution structure (panel set 3 of 4). Figure S19. Continuation of model-specific SHAP profiles for models with the most contrasting or divergent wavelength-attribution patterns (panel set 4 of 4). Table S1. Complete list of the thirty common bean landraces (*Phaseolus vulgaris*) and the two non-*Phaseolus* outgroup legumes (*Vigna angularis* and *Cajanus cajan*), with accession codes and seed phenotype descriptions used in this study. Descriptions reflect recorded cultivar names or batch-level visual phenotypes for which no standard description was available. Table S2. Wavelengths selected by supervised ranking, with ReliefF score, ANOVA eta^2^, mean absolute SHAP value across models, coefficient of variation, and edge-band status. Table S3. Micro- and macroaveraged one-versus-rest ROC-AUC and average precision (AP) summaries for the principal reduced-band benchmark models evaluated on the held-out test set.
