# Supplementary material for: VIS–NIR–SWIR Hyperspectral Imaging and Advanced Machine and Deep Learning Algorithms for a Controlled Benchmark of Bean Seed Identification and Classification
Source: Plants (Basel). 2026 Mar 18;15(6):933. doi: 10.3390/plants15060933 (PMC13030257; doi:10.3390/plants15060933)
Supplement: Supplementary file 1 [file plants-15-00933-s001.zip › plants-4162527-supplementary.pdf]

plants

MDPI

Supplementary files

VIS–NIR–SWIR Hyperspectral Imaging and Advanced Machine and Deep Learning Algorithms for a Controlled Benchmark of Bean Seed Identification and Classification

Renan Falcioni [1,*], Nicole Ghinzelli Vedana [1], Caio Almeida de Oliveira [1], João Vitor Ferreira Gonçalves [1], Marcelo Luiz Chicati [1], José Alexandre M. Demattê [2] and Marcos Rafael Nanni [1]

[1] Graduate Program in Agronomy, State University of Maringá, Av. Colombo 5790, Maringá 87020-900, Paraná, Brazil; pg405864@uem.br (N.G.V.); pg55482@uem.br (C.A.d.O.); pg55494@uem.br (J.V.F.G.); mlchicati@uem.br (M.L.C.); mrnanni@uem.br (M.R.N.)
[2] Department of Soil Science, Luiz de Queiroz College of Agriculture, University of São Paulo, Av. Pádua Dias 11, Piracicaba 13418-260, São Paulo, Brazil; jamdemat@usp.br
* Correspondence: renanfalcioni@gmail.com

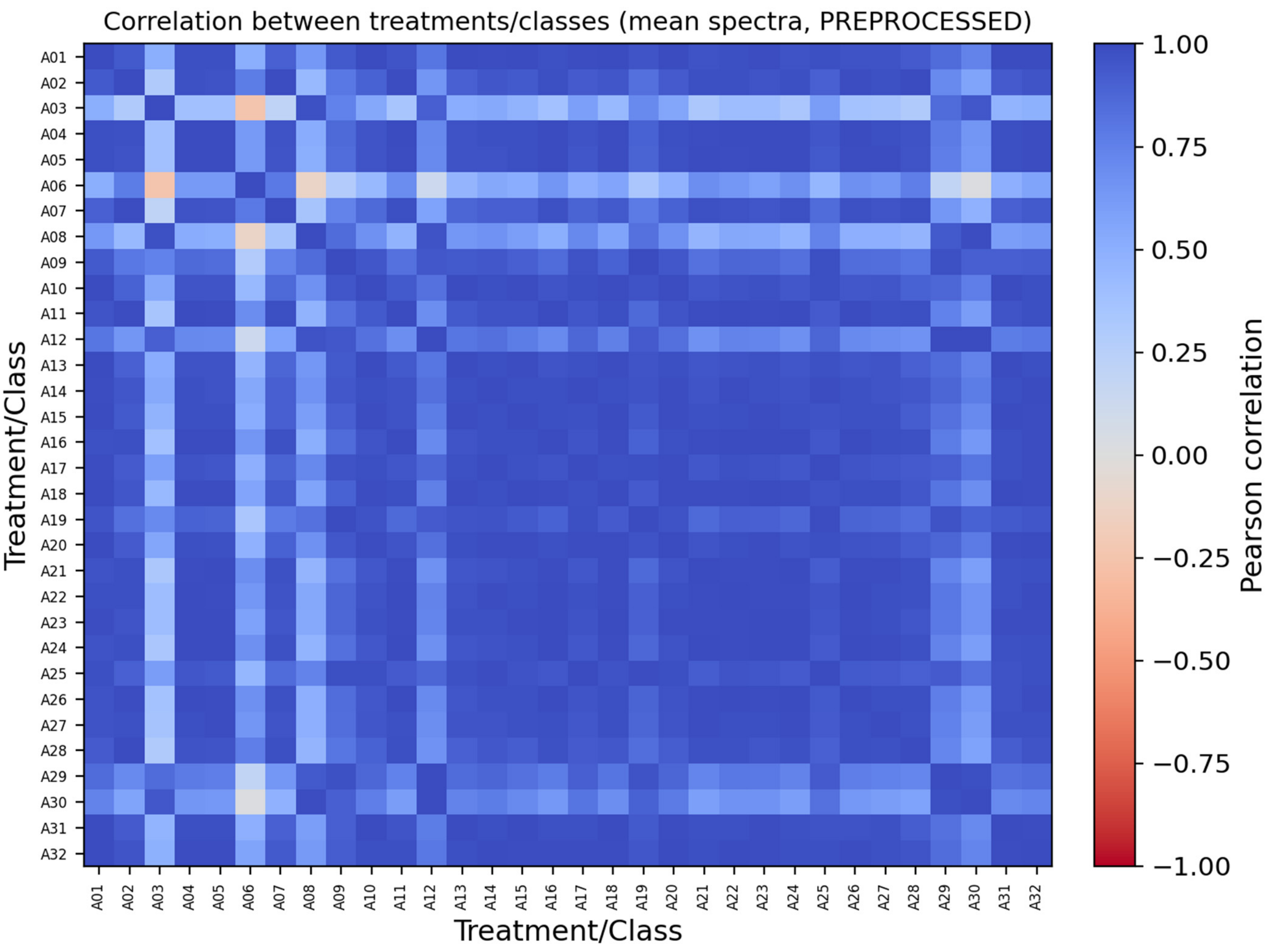

Figure S1. Correlation matrix of the accession-mean spectra across A01–A32, showing pairwise Pearson correlation coefficients among the preprocessed class means. The plot summarises the spectral similarity across accessions and highlights both closely related and more distinct spectral neighbourhoods within the dataset.

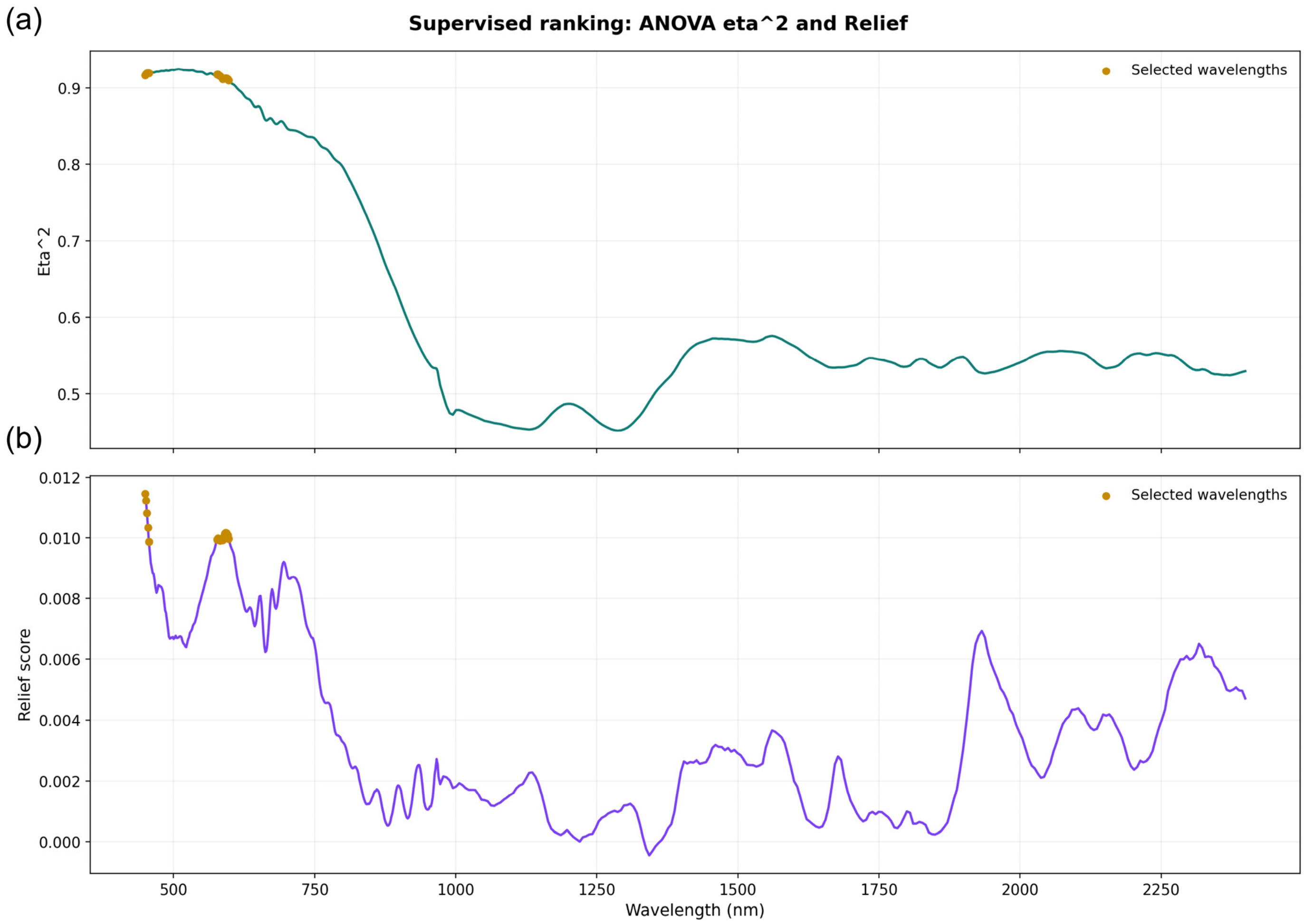

Figure S2. Supervised wavelength ranking based on ANOVA eta² and ReliefF, with the selected wavelengths indicated on both profiles to compare the global between-class effect size and supervised local feature relevance.

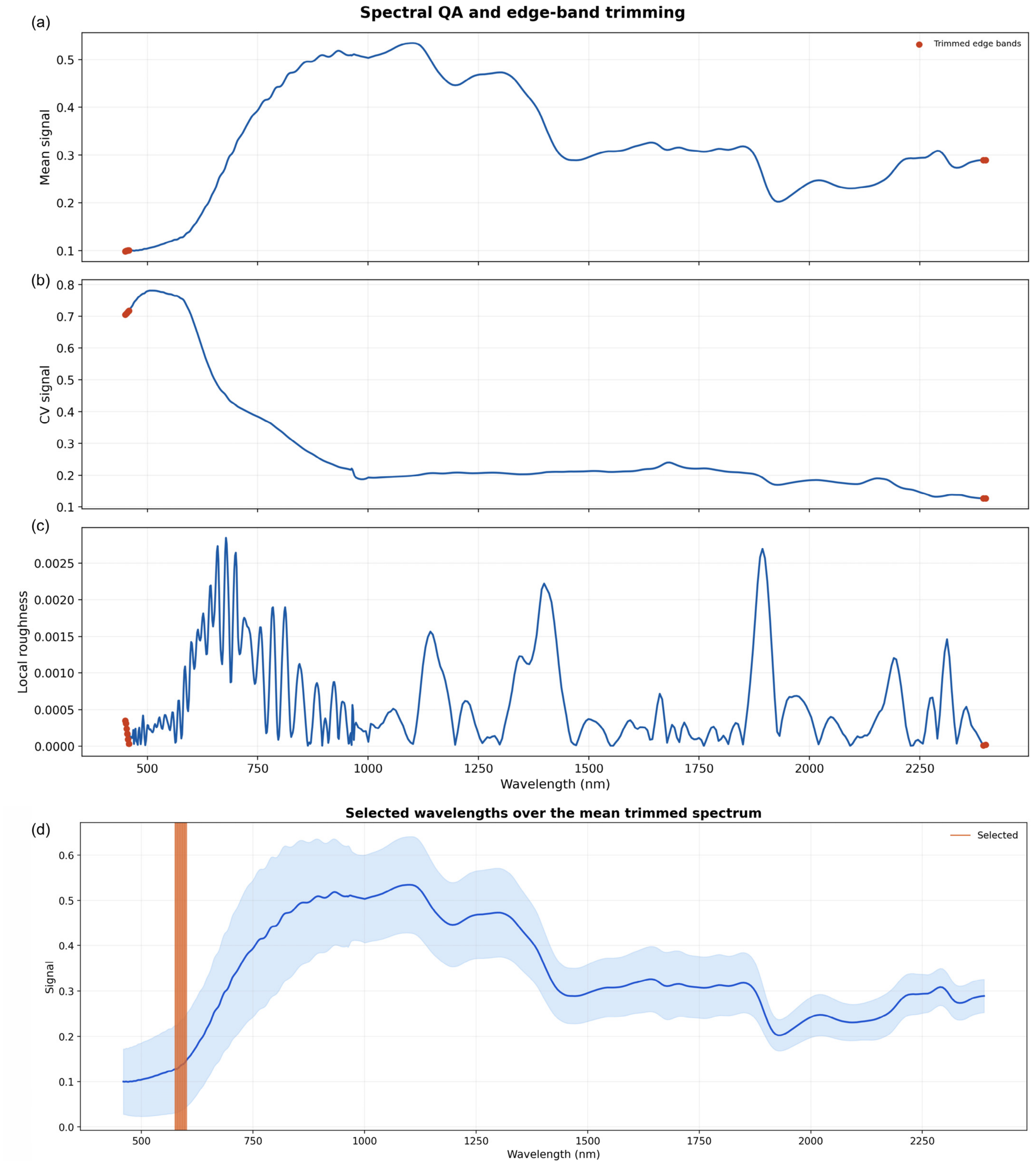

Figure S3. Spectral quality control and edge band trimming. Panels show the mean signal, coefficient of variation, local roughness, and placement of selected wavelengths over the trimmed mean spectrum, providing the rationale for removing unstable spectral-edge regions.

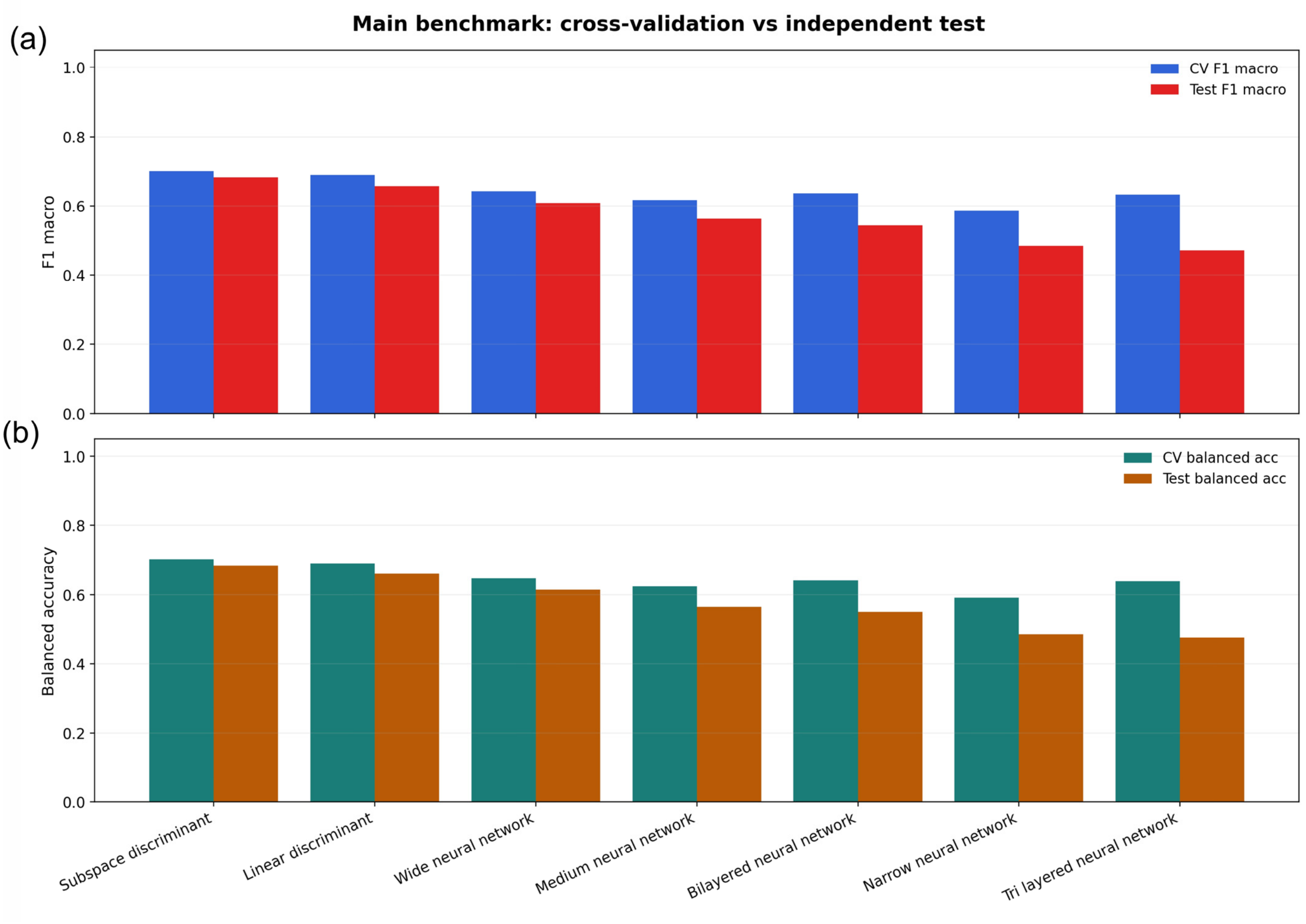

Figure S4. Agreement between cross-validation and independent-test performance in the main benchmark. (a) Macro-F1 score and (b) balanced accuracy for the best-performing classical models, highlighting the generalisation gap between development and held-out evaluation.

https://doi.org/10.3390/plants15060933

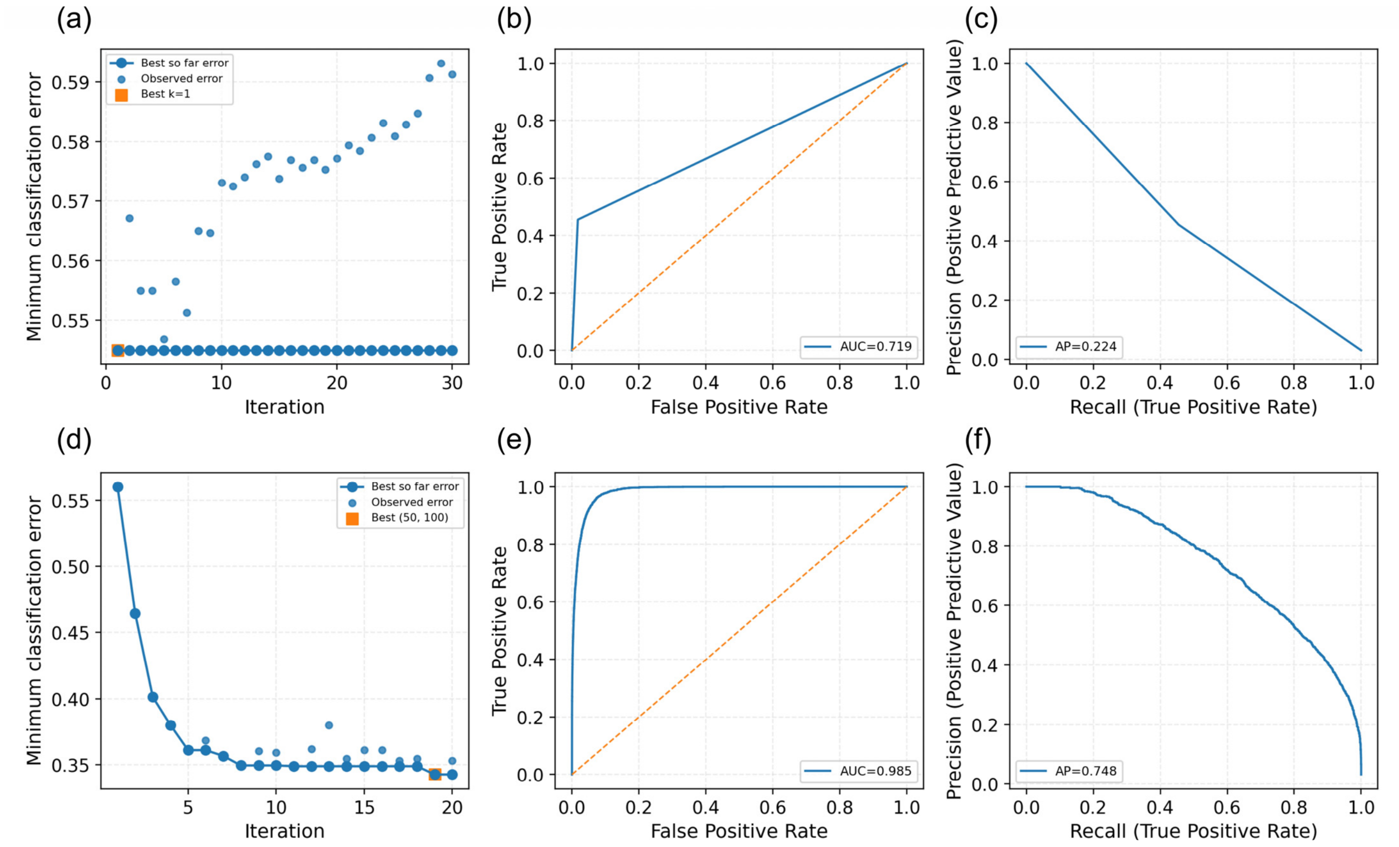

Figure S5. Internal optimisation diagnostics and ranking-quality summaries for representative baseline workflows. Panels show validation-error trajectories during model tuning and the corresponding ROC and precision–recall curves used to summarise ranking performance.

https://doi.org/10.3390/plants15060933

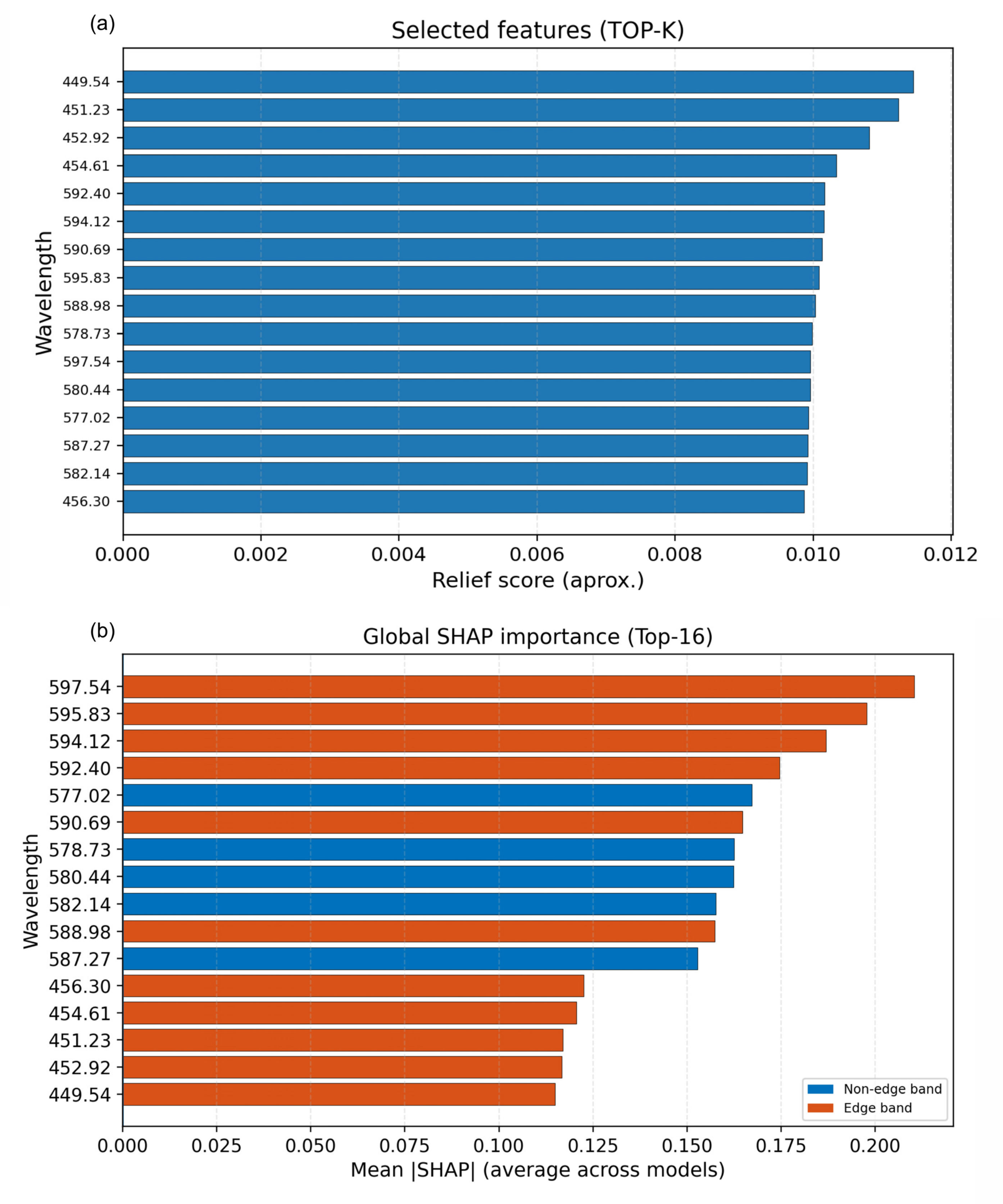

Figure S6. Top-k wavelength selection and global SHAP attribution for the reduced-band configuration. (a) Relief-based ranking of the selected wavelengths. (b) Mean absolute SHAP importance across models, contrasting edge bands and interior bands.

https://doi.org/10.3390/plants15060933

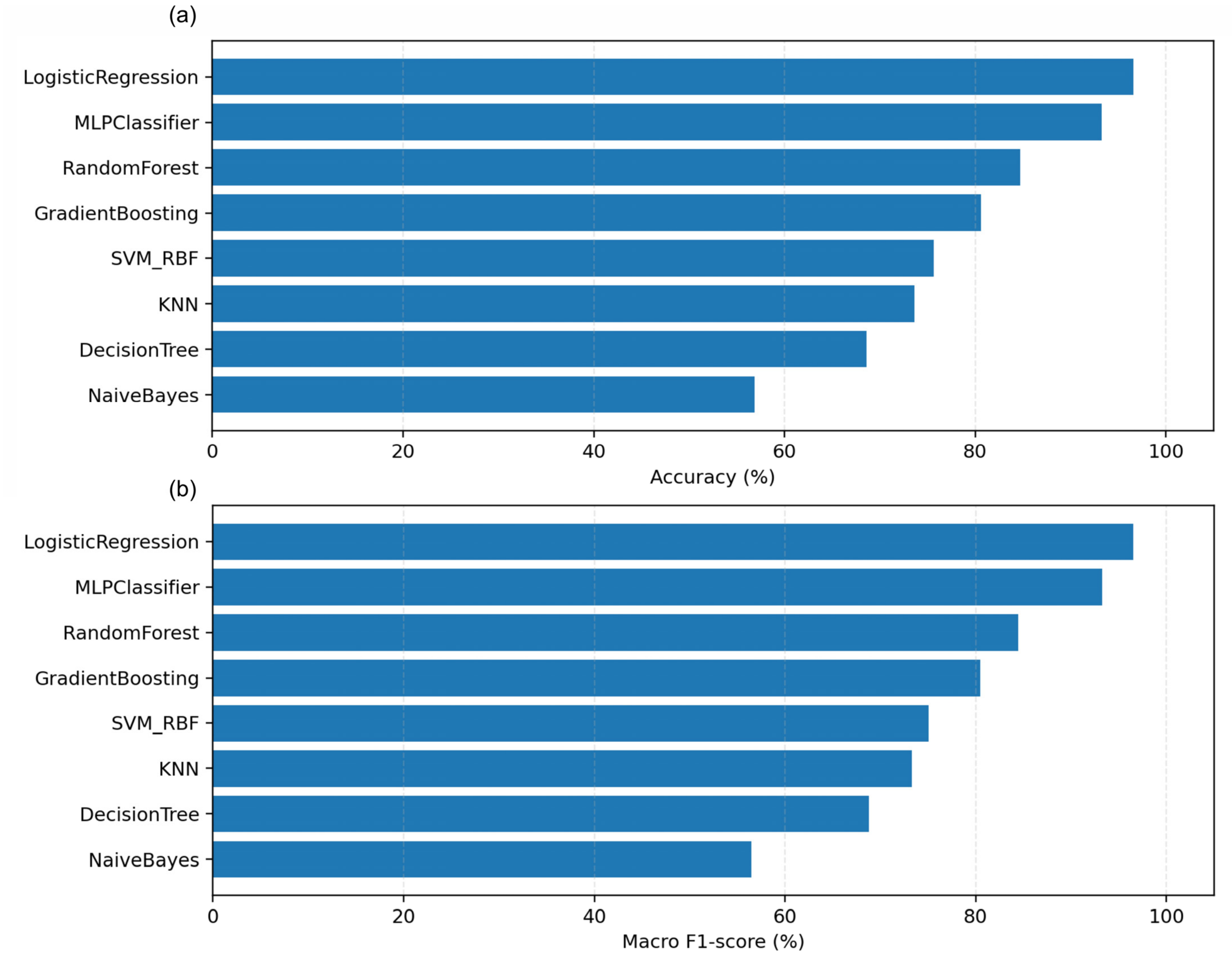

Figure S7. (a) Accuracy and **(b)** macro-F1 score of the additional baseline classifiers evaluated on the independent test set.

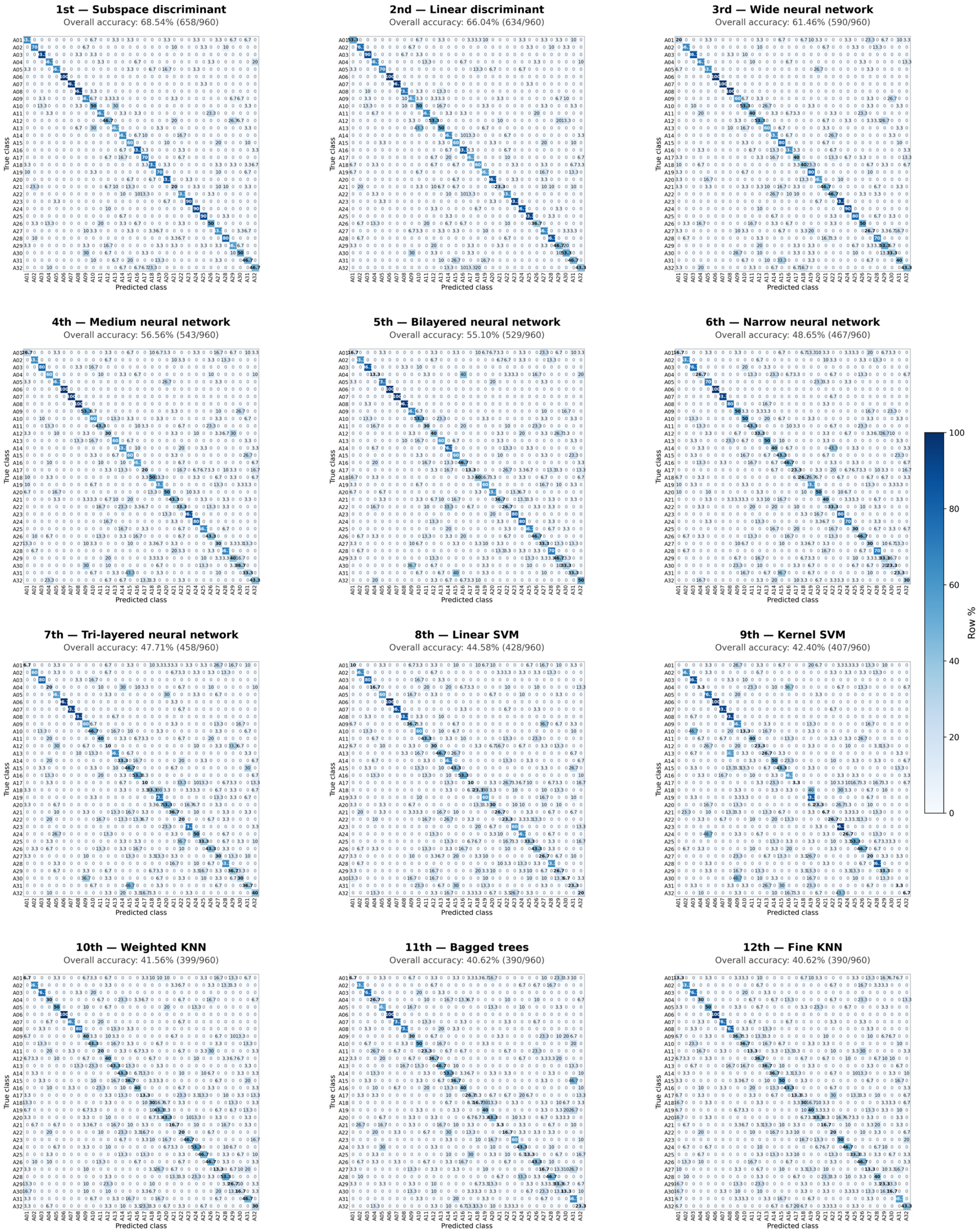

Figure S8. Row-normalised confusion matrices for the 12 highest-ranking classifiers in the benchmark, ordered from best to worst overall accuracy. The percentages are shown in cells to facilitate comparisons of class-specific recall and dominant confusion patterns.

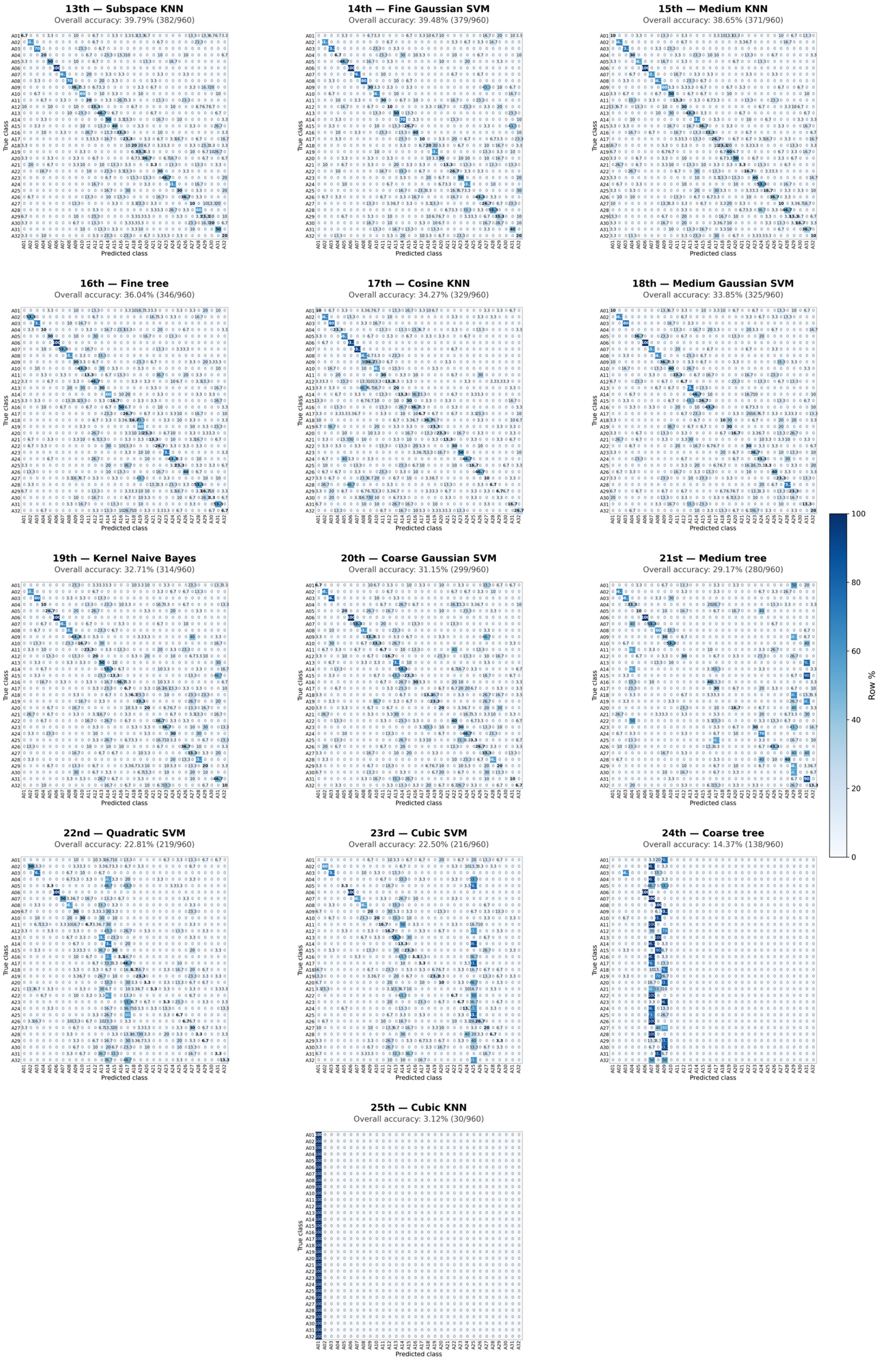

Figure S9. Row-normalised confusion matrices for classifiers ranked 13th to 25th, and the benchmark ordering is shown in Figure S8. Percentages are displayed in cells to reveal how misclassification patterns change in the lower-performing models.

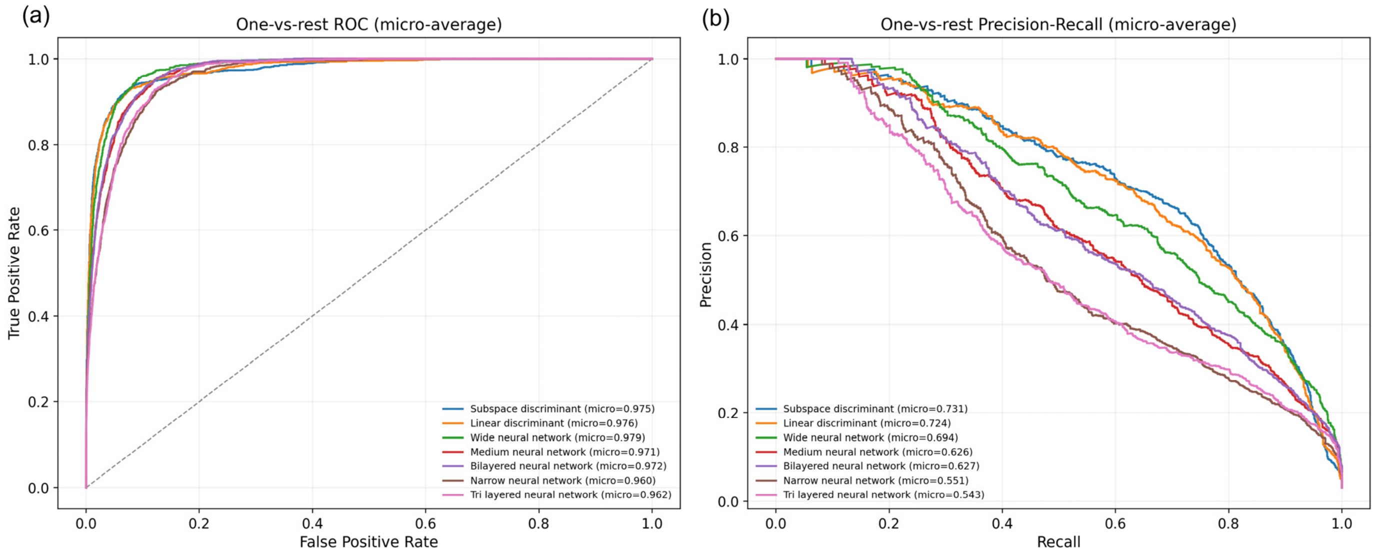

Figure S10. Microaveraged one-versus-rest ROC and precision–recall curves for the main benchmark models, summarising the ranking performance across all classes.

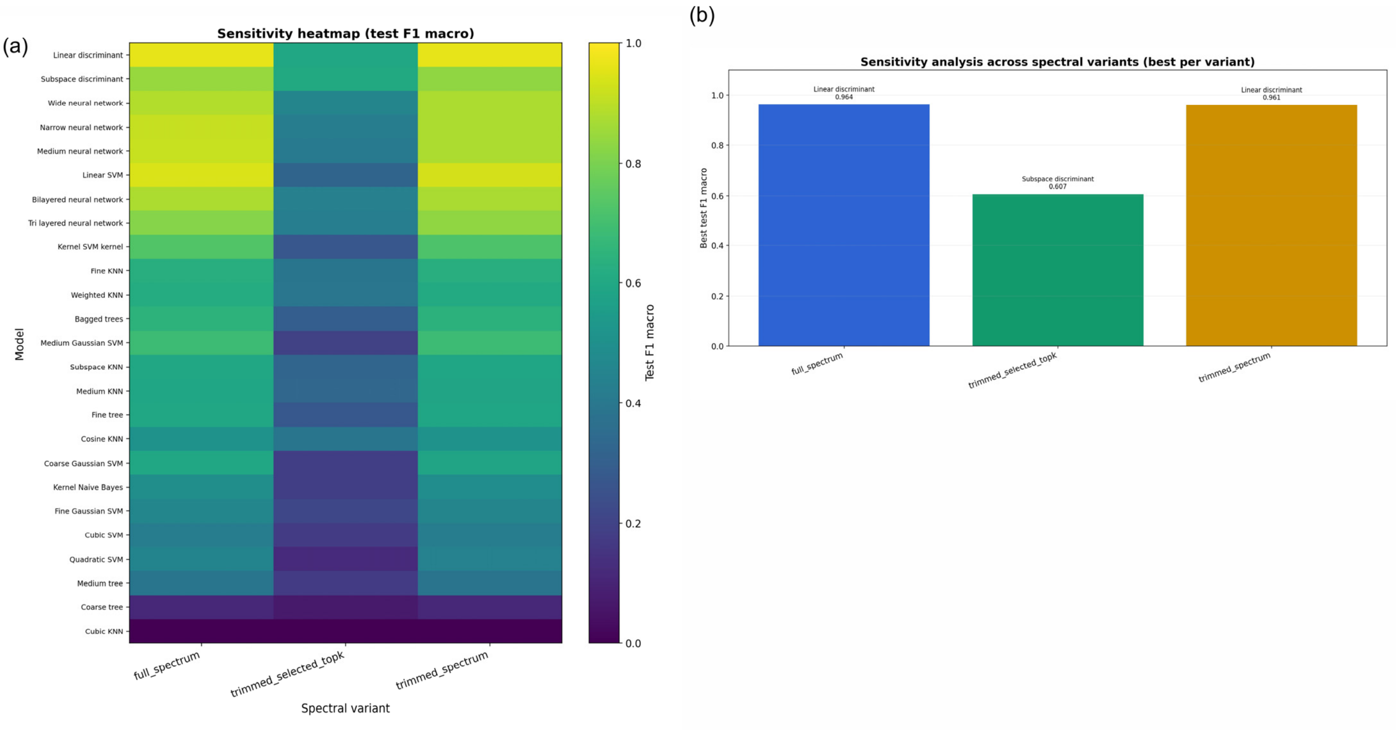

Figure S11. Heatmap summary of model sensitivity across spectral variants and comparison of the best score obtained within each variant. **(a)** The heatmap shows the macro-F1 test results by model and spectral representation, whereas panel **(b)** shows the top-performing model for each variant.

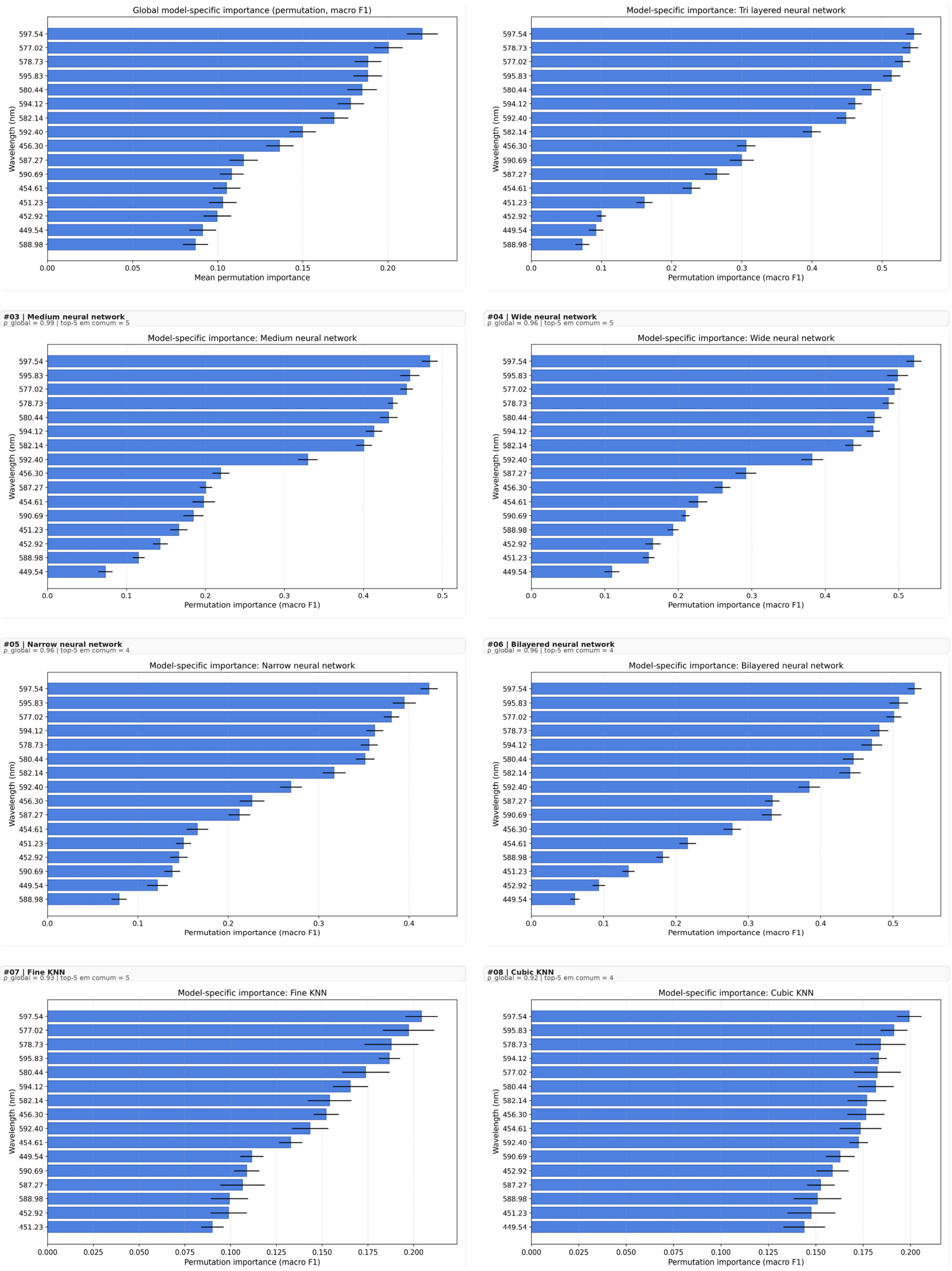

Figure S12. Model-specific permutation-importance profiles for the global reference and the models with the closest agreement with the global wavelength ranking (panel set 1 of 4). The bars represent the importance of macro-F1-based permutations across the selected wavelengths.

https://doi.org/10.3390/plants15060933

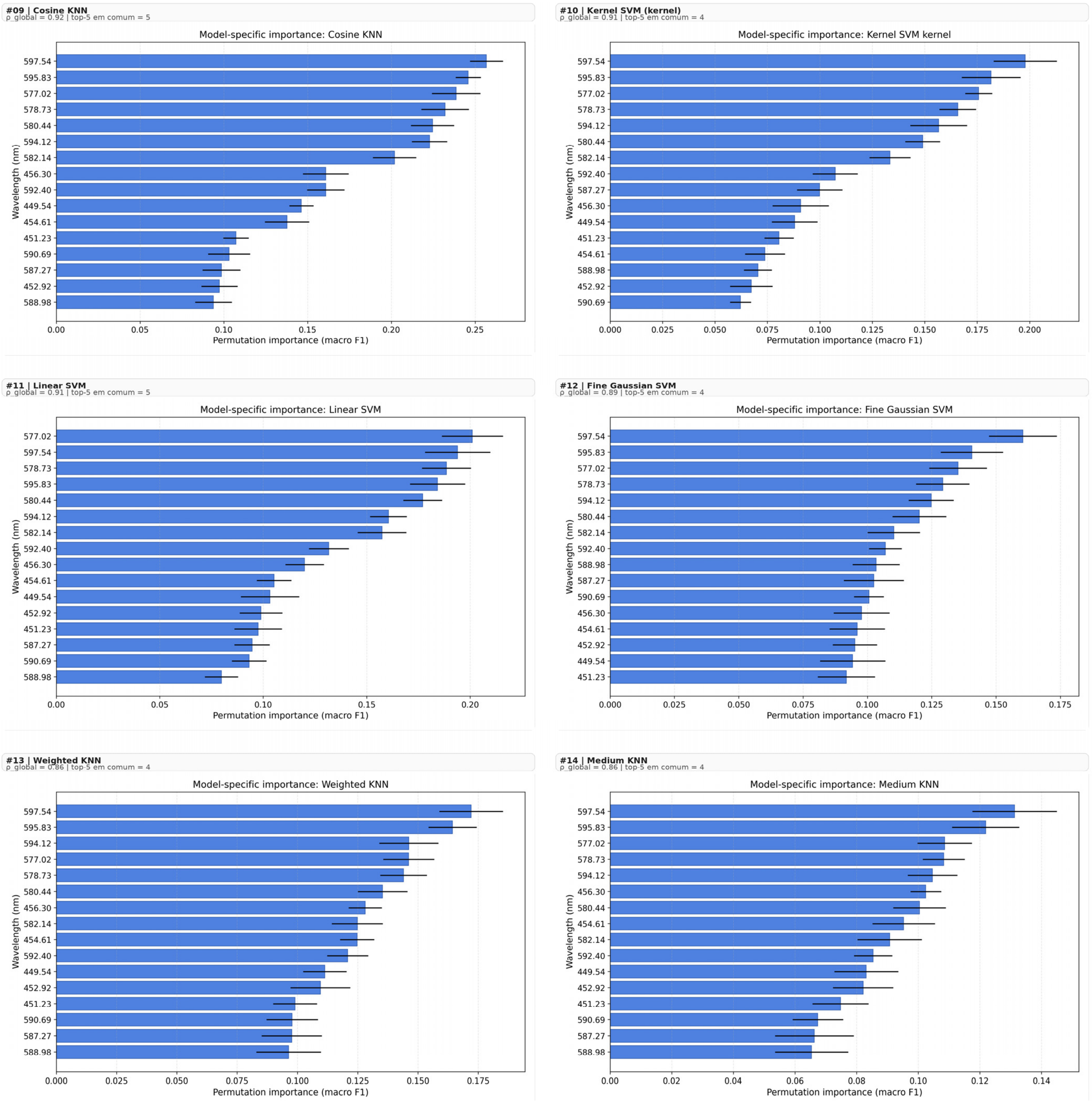

Figure S13. Continuation of model-specific permutation-importance profiles for models with high concordance with the global wavelength ranking (panel set 2 of 4).

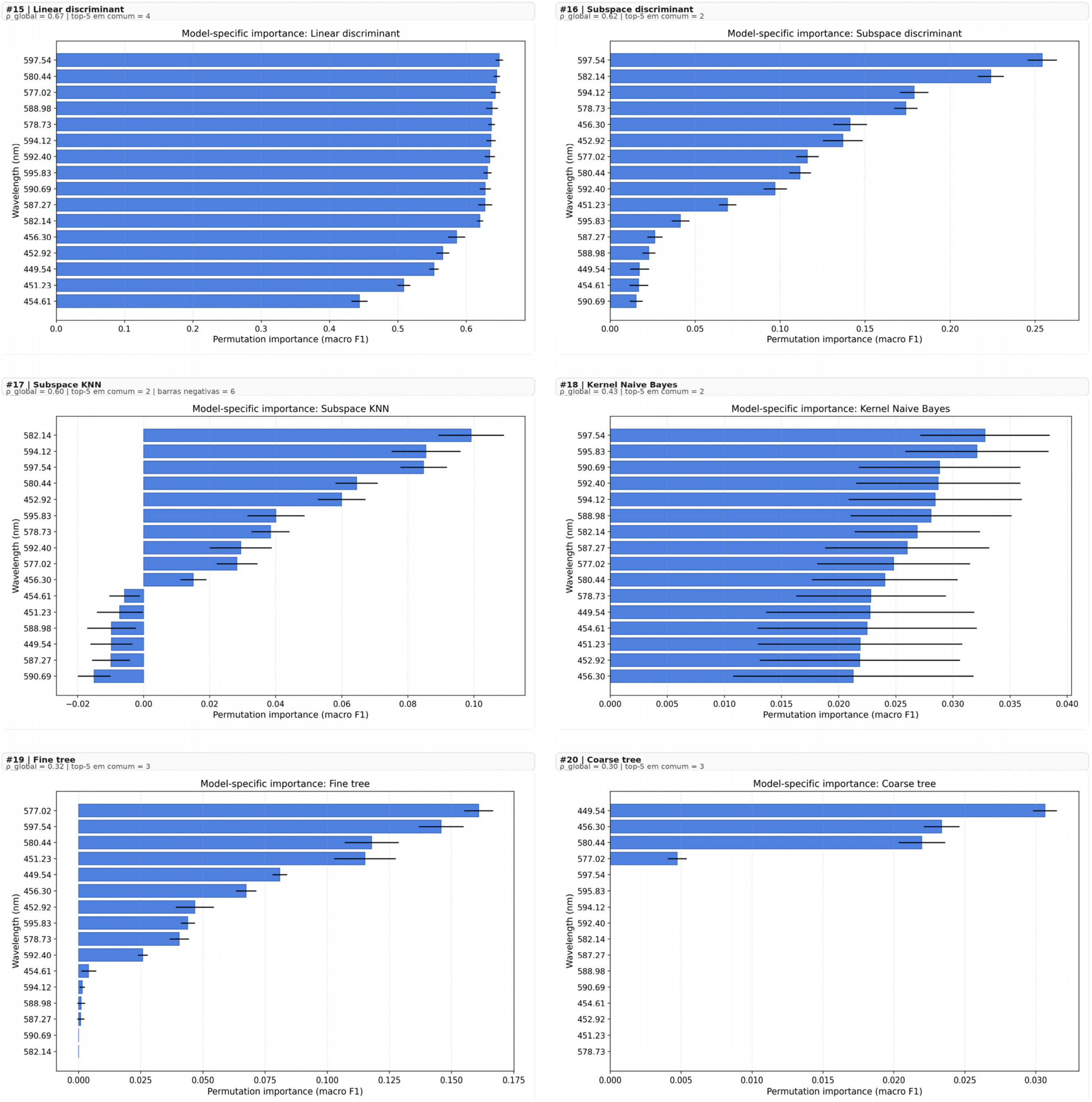

Figure S14. Continuation of model-specific permutation-importance profiles for models with intermediate concordance and hybrid wavelength-importance profiles (panel set 3 of 4).

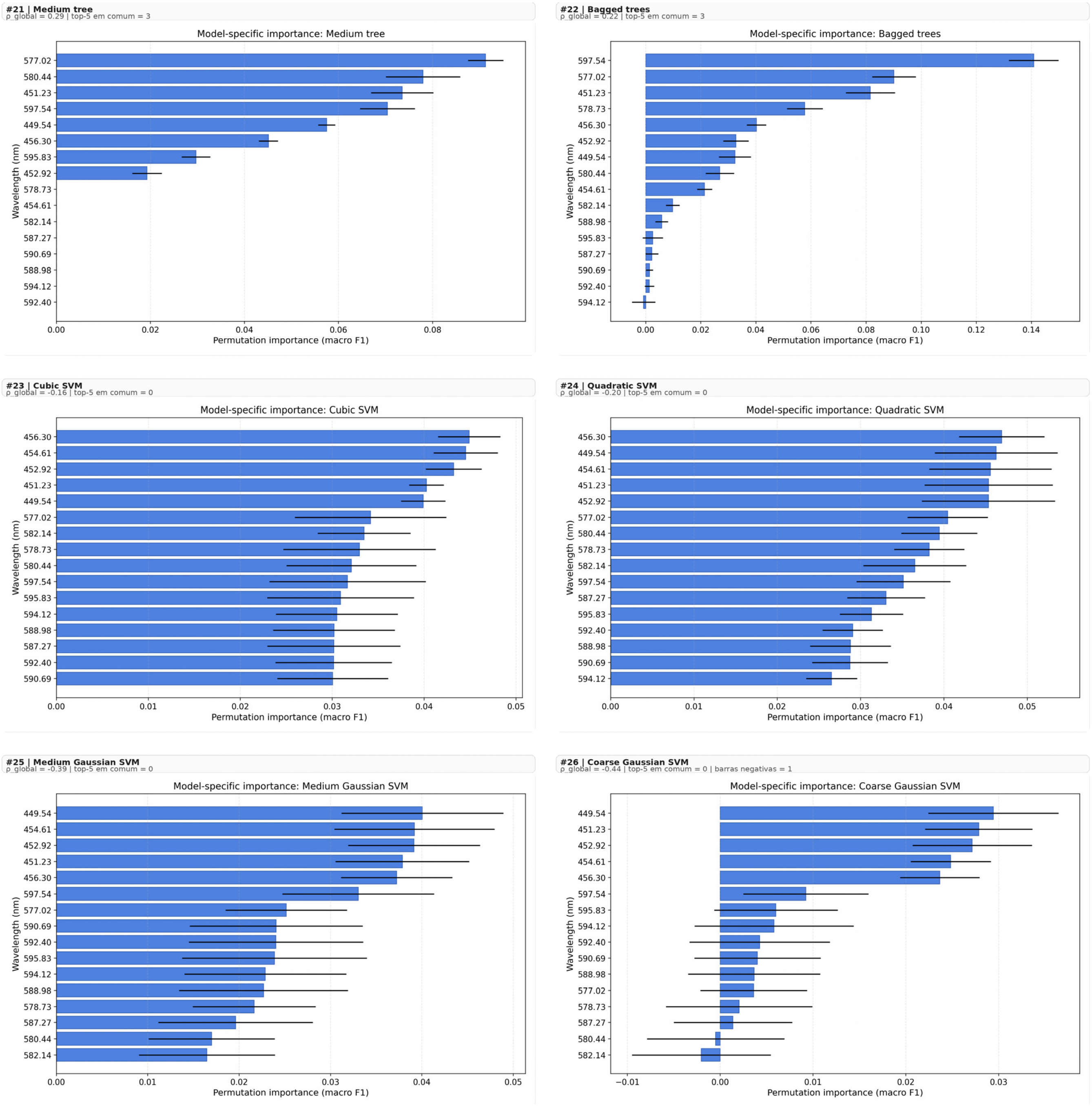

Figure S15. Continuation of model-specific permutation-importance profiles for models with the most divergent, sparse, or contrasting wavelength-importance patterns (panel set 4 of 4).

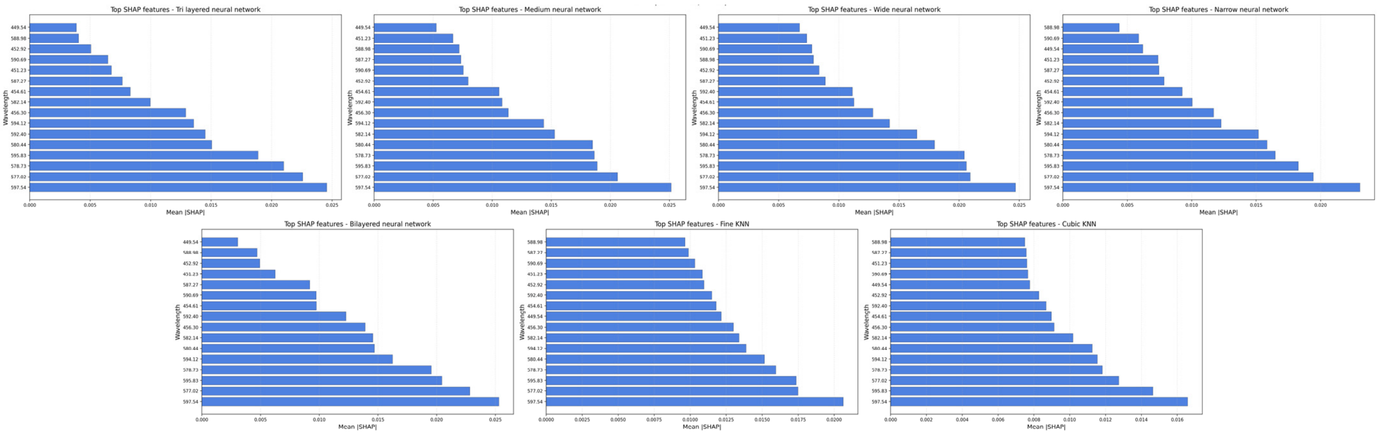

Figure S16. Model-specific SHAP profiles for the models most aligned with the global importance pattern (panel set 1 of 4). The bars represent the mean absolute SHAP values across the selected wavelengths.

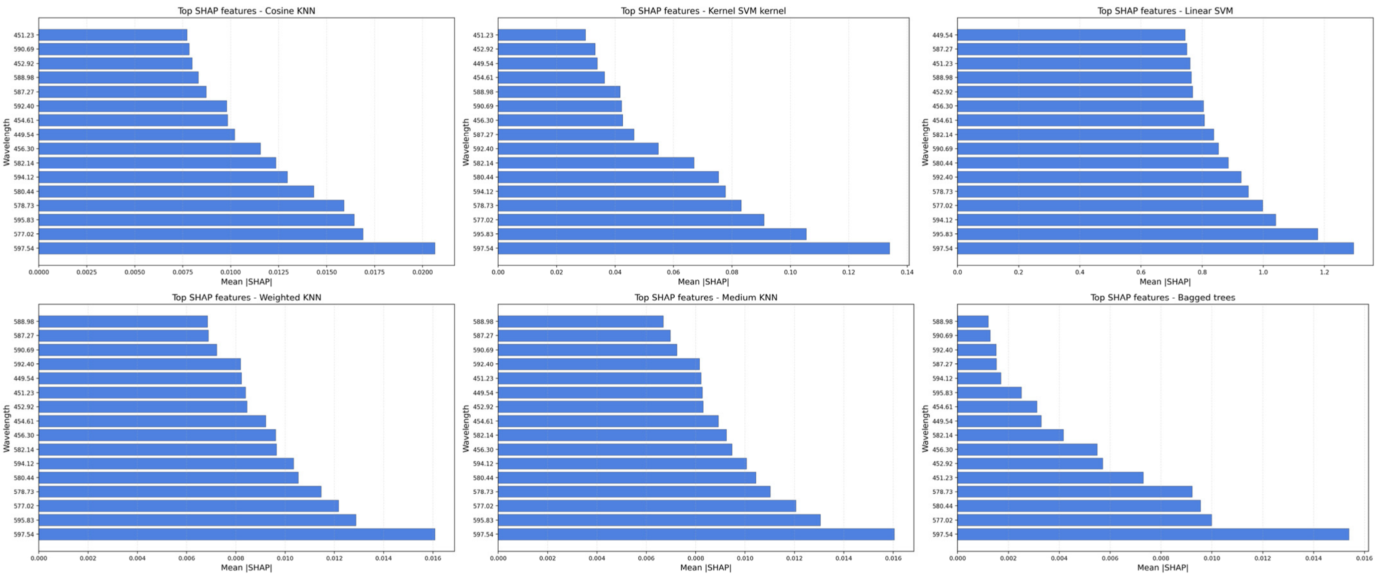

Figure S17. The continuation of model-specific SHAP profiles for models with high, although not identical, agreement with the global importance pattern (panel set 2 of 4).

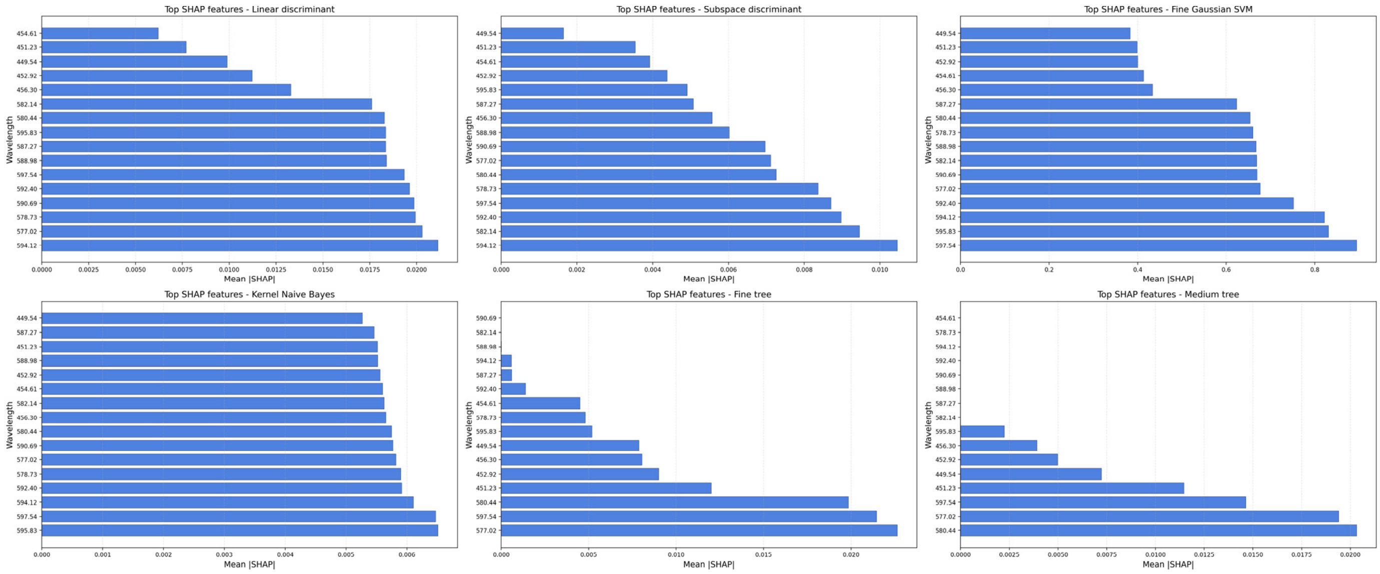

Figure S18. Continuation of model-specific SHAP profiles for models with intermediate selectivity and partially divergent attribution structure (panel set 3 of 4).

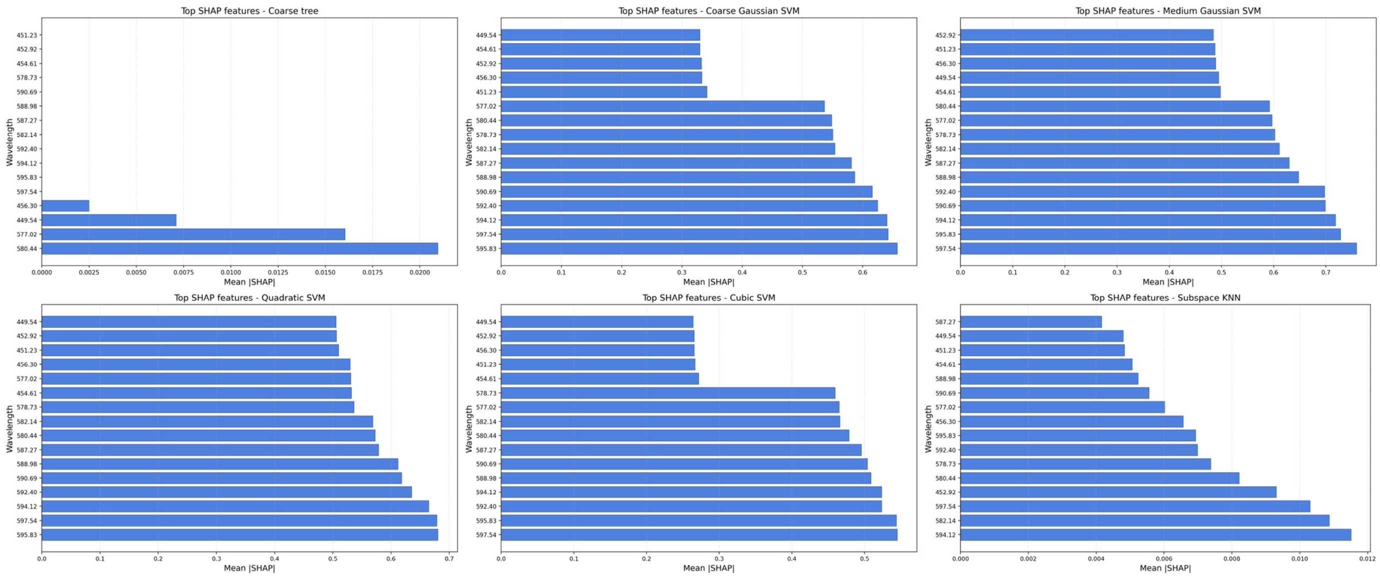

Figure S19. Continuation of model-specific SHAP profiles for models with the most contrasting or divergent wavelength-attribution patterns (panel set 4 of 4).

Table S1. Complete list of the thirty common bean landraces (*Phaseolus vulgaris*) and the two non-*Phaseolus* outgroup legumes (*Vigna angularis* and *Cajanus cajan*), with accession codes and seed phenotype descriptions used in this study. Descriptions reflect recorded cultivar names or batch-level visual phenotypes for which no standard description was available.

**Abbreviation**[1]	**Accession name**[2]	**Characteristics of beans**[3]
A01	Feijão Amendoim Roxo	Purple/violet seed coat; elongated 'peanut-type' seeds; uniform pattern.
A02	Feijão Carioca	Cream/beige seed coat with brown stripes (carioca type); kidney-shaped seeds; medium size.
A03	Feijão Preto Gigante	Black seed coat; large seeds; uniform pattern.
A04	Feijão Branco Manteiga	White/pale cream seed coat (butter type); large seeds; uniform pattern.
A05	Feijão Rosinha	Pink seed coat; medium-sized seeds; uniform pattern.
A06	Feijão Branco Gigante	White seed coat; very large seeds; uniform pattern.
A07	Feijão Canário	Canary-yellow seed coat; medium-sized seeds; uniform pattern.
A08	Feijão Preto Mocotó	Black seed coat; medium-sized seeds; uniform pattern.
A09	Feijão Verde	Green seed coat (olive to light green); medium-sized seeds; uniform pattern.
A10	Feijão Vermelho Bolinha	Red seed coat; small, rounded ('little ball') seeds; uniform pattern.
A11	Feijão Branco Bolinha	White seed coat; small, rounded seeds; uniform pattern.
A12	Feijão Mouro Gigante	Dark seed coat (mouro: dark brown to black); large seeds; uniform to slightly mottled pattern.
A13	Feijão Roxinho	Purplish-red seed coat; medium-sized seeds; uniform pattern.
A14	Feijão Creme	Cream/light beige seed coat; medium-sized seeds; uniform pattern.
A15	Feijão Vermelho Gigante	Red seed coat; large seeds; uniform pattern.
A16	Feijão Jalo	Golden-yellow seed coat (jalo type); large seeds; uniform pattern.
A17	Feijão Amendoim Bege	Beige seed coat; elongated 'peanut-type' seeds; uniform pattern.
A18	Feijão Boreal Rosa	Pink seed coat; medium-sized seeds; uniform pattern.
A19	Feijão Chocolate	Chocolate-brown seed coat; medium-sized seeds; uniform pattern.
A20	Feijão Amendoim Rosa	Pink seed coat; elongated 'peanut-type' seeds; uniform pattern.
A21	Feijão Olho de Pombo	Cream seed coat with a dark hilum ('eye'); bicolour 'pigeon-eye' pattern; small to medium seeds.

https://doi.org/10.3390/plants15060933

A22	Feijão Bico de Ouro	Yellow/golden seed coat (‘bico-de-ouro’ type); medium-sized seeds; uniform pattern.
A23	Feijão Amendoim	Beige to light-brown seed coat; elongated ‘peanut-type’ seeds; uniform pattern.
A24	Feijão Ovo de Tito-Tico	Light-coloured seed coat with contrasting speckles/blotches (‘tico-tico egg’ type); small, oval seeds.
A25	Feijão Amendoim Verde	Green seed coat; elongated ‘peanut-type’ seeds; uniform pattern.
A26	Feijão Rajado	Light seed coat with darker streaks/blotches (striped/mottled); medium-sized seeds; variegated pattern.
A27	Feijão Azaki	Azuki (*Vigna angularis*): typically uniform red seed coat; small seeds.
A28	Feijão Guandu	Pigeon pea (*Cajanus cajan*): rounded seeds; cream to brown seed coat; typically uniform pattern.
A29	Feijão Mouro	Dark seed coat (mouro); small to medium seeds; uniform pattern.
A30	Feijão Mancá	Local landrace (‘Mancá’): based on the observed seed colour/pattern of the batch (no standard description available).
A31	Feijão Vermelho	Red seed coat; medium-sized seeds; uniform pattern.
A32	Feijão Rapa Cuiua	Rapa Cuia/Rapa Cuiua (*Phaseolus vulgaris*): traditional landrace; associated with dark/black beans; dark seeds.

[1]Abbreviation (ID): identification code used to trace accessions through spectral extraction and modelling. [2]Accession name: common/popular name of each accession. [3]Characteristics of beans: morphological description of the analysed accessions on the basis of the images of the batches.

Table S2. Wavelengths selected by supervised ranking, with ReliefF score, ANOVA eta², mean absolute SHAP value across models, coefficient of variation, and edge-band status.

Wavelength (nm)	Region	Relief score	ANOVA eta²	Mean |SHAP|	CV (%)	Edge band
449.54	VIS blue–green (400-500)	0.0115	0.9169	0.1150	70.51	Yes
451.23	VIS blue–green (400-500)	0.0112	0.9188	0.1170	70.69	Yes
452.92	VIS blue–green (400-500)	0.0108	0.9196	0.1167	70.92	Yes
454.61	VIS blue–green (400-500)	0.0103	0.9198	0.1206	71.19	Yes
456.30	VIS blue–green (400-500)	0.0099	0.9199	0.1226	71.48	Yes
577.02	VIS red (500-700)	0.0099	0.9181	0.1672	75.61	No
578.73	VIS red (500-700)	0.0100	0.9180	0.1624	75.51	No
580.44	VIS red (500-700)	0.0100	0.9173	0.1624	75.36	No
582.14	VIS red (500-700)	0.0099	0.9159	0.1577	75.12	No
587.27	VIS red (500-700)	0.0099	0.9122	0.1528	73.84	No
588.98	VIS red (500-700)	0.0100	0.9124	0.1573	73.37	No
590.69	VIS red (500-700)	0.0101	0.9128	0.1648	72.91	No
592.40	VIS red (500-700)	0.0102	0.9131	0.1746	72.45	No
594.12	VIS red (500-700)	0.0102	0.9129	0.1869	71.99	No
595.83	VIS red (500-700)	0.0101	0.9119	0.1977	71.48	No
597.54	VIS red (500-700)	0.0100	0.9104	0.2103	70.90	No

https://doi.org/10.3390/plants15060933

Table S3. Micro- and macroaveraged one-versus-rest ROC-AUC and average-precision (AP) summaries for the principal reduced-band benchmark models evaluated on the held-out test set.

Model	ROC-AUC (micro)	ROC-AUC (macro)	AP (micro)	AP (macro)
Subspace discriminant	0.975	0.978	0.731	0.736
Linear discriminant	0.976	0.978	0.724	0.705
Wide neural network	0.979	0.973	0.694	0.650
Medium neural network	0.971	0.965	0.626	0.587
Bilayered neural network	0.972	0.965	0.627	0.591
Narrow neural network	0.960	0.953	0.551	0.518
Tri layered neural network	0.962	0.954	0.543	0.505

https://doi.org/10.3390/plants15060933
